# Distinct spatial organization governs oral mucosal immunity

**DOI:** 10.1038/s41590-025-02398-y

**Published:** 2026-02-09

**Authors:** Vasileios I. Theofilou, David Fraser, Eleni Kanasi, Laurie Brenchley, Teresa Greenwell-Wild, Emmanuel E. Adade, Alex M. Valm, Iyadh Douagi, Yasmine Belkaid, Duy T. Tran, Drake W. Williams, Niki M. Moutsopoulos

**Affiliations:** 1 Human Barrier Immunity Section, Laboratory of Host Immunity and Microbiome, National Institute of Allergy and Infectious Diseases (NIAID), National Institutes of Health (NIH), Bethesda, MD USA; 2 Department of Oncology and Diagnostic Sciences, School of Dentistry, University of Maryland, Baltimore, MD USA; 3 National Institute of Dental and Craniofacial Research, NIH, Bethesda, MD USA; 4 Department of Biological Sciences, University at Albany State University of New York, Albany, NY USA; 5 NIH Center for Human Immunology, Autoimmunity, and Inflammation (CHI), NIAID, NIH, Bethesda, MD USA; 6 Metaorganism Unit, Department of Immunology, Institut Pasteur, Paris, France; 7 National Eye Institute, NIH, Bethesda, MD USA; 8 Department of Diagnostic and Biological Sciences, University of Minnesota, Minneapolis, MN USA; 9 Center for Immunology, University of Minnesota, Minneapolis, MN USA; 10 Masonic Cancer Center, University of Minnesota, Minneapolis, MN USA

**Keywords:** Mucosal immunology, Inflammatory diseases

## Abstract

Immune responsiveness at barrier surfaces is tailored to the exposures of each tissue. In the oral mucosa, mechanisms by which a permeable epithelium coexists with diverse microbiota and maintains integrity during inflammatory pathology remain poorly understood. We compile a multiomics spatial map of this exposed mucosal microenvironment and uncover remarkable immune zonation with organization that is preserved even during inflammatory disease. At the tooth interface, we identify a dynamic epithelium underlined by a layer of neutrophils and a zone of antigen-presenting cell–lymphocyte aggregates. During disease, inflammatory zones expand and organize into immature tertiary lymphoid structures, suggesting local antibody production. Location-specific transcriptomes support a role for the stromal compartment in the spatial organization of immunity. This preserved immune zonation meets the demands for continuous protection of this vulnerable interface and suggests unique tissue-specific wiring of immunity at the human oral mucosal barrier.

## Abstract (web summary)

Moutsopoulos, Williams and colleagues show oral mucosal barrier tissues exhibit dynamic zonal immune cell environments, including tonic inflammatory features in healthy individuals and development of tertiary lymphoid structures with the potential to support local antibody production in periodontitis.

## Main

Barrier sites, such as the skin, gastrointestinal tract and respiratory tract, are constantly exposed to the outside environment. Their role is to both provide physical separation from the outside world and integrate complex environmental signals, offering surveillance and protection from external insults, while ignoring or actively tolerating innocuous antigens and commensals^1,2^. In recent years there has been increased interest in understanding the intricacies of tissue-specific immunity at barrier sites in both health and disease, particularly through the use of experimental models^2,3^; however, human barrier immunity is highly complex and remains incompletely understood.

A major question of interest is how the most exposed and vulnerable sites within barrier tissues develop sophisticated and dynamic immunological networks to maintain balance with the environment. For instance, in the skin, areas with high external exposure, such as hair follicles, develop local immunological hubs that mediate active immunoregulation^4^. Similarly, in the small intestine, where the epithelium is particularly thin, complex intra- and subepithelial immunological networks, including subepithelial lymphoid structures (Peyer’s patches), orchestrate regional immunity^5^.

In this regard, the oral mucosa is a particularly exposed barrier surface. It is a site of first encounters for the host, as food, commensals and airborne elements enter the human body through this portal. Unlike the skin, most of the oral mucosal surface has minimal keratinization and lacks submucosal lymphoid tissues, despite harboring rich and diverse commensal microbial communities^6^. Yet, infection of the oral mucosa is rare in immunocompetent individuals and wound healing is optimal with lack of scarring or infection^7^. Understanding how the local immune system maintains homeostasis in health and controls widespread systemic microbial translocation and infection, is a key question in human physiology with relevance across barrier tissues.

Dissecting the balance between environment and host is particularly relevant at the most vulnerable site of the oral mucosa, the tooth-associated epithelium (TAE)^6^. The TAE (encompassing the junctional and sulcular epithelia) is thin, nonkeratinized and highly permeable. It is located adjacent to the tooth-associated microbiome and is subject to constant mechanical damage from chewing and hygiene^8^. Despite this constant mechanical and microbial insult, the oral barrier maintains a state of homeostasis. This resilience persists even in periodontitis, a common human disease where the local microbial biomass becomes tremendously increased and dysbiotic^9–11^ and the barrier epithelium undergoes constant cycles of damage and repair. However, both tissue-invasive infection and systemic overt bacteremia rarely occur despite transient systemic translocation of periodontal microbiota^12^. Furthermore, signs of defective wound healing or scarring are very uncommon in this environment, despite constant triggering^7^. This suggests a unique wiring of the local immune response toward protection from invasive infection, while conditioned for optimal wound healing.

To gain insights into this unique human barrier, we applied an integrated multiomics approach, expanding upon previous single-cell sequencing studies^13,14^ by adding spatial context at cellular resolution in health and disease. Our approach uncovers a previously unappreciated inflammatory cell organization, reveals location-specific functionality of the epithelial and stromal compartments in orchestrating local immunity, and opens new avenues toward understanding human mucosal inflammatory disease.

## Results

### Integrated multiomics atlas of human gingiva

**Fig. 1:**
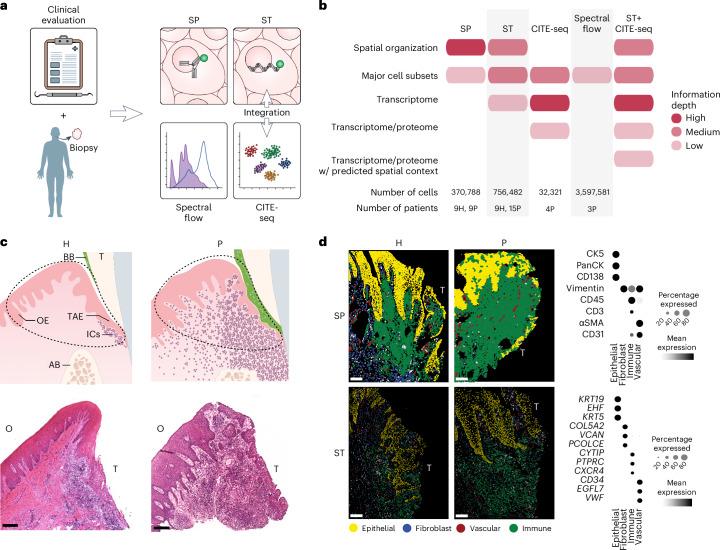
An integrated multiomics atlas of the gingival mucosal barrier in health and periodontitis. **a**, Biopsies of oral mucosa (gingiva) were obtained from systemically healthy volunteers with oral health (H) or periodontitis (P) and processed for spatial and single-cell profiling assays. **b**, Summary of key strengths of each approach, number of analyzed cells and individuals included. **c**, Schematic representation of gingival tissue histology and adjacent structures in health and periodontitis (top). Dotted lines depict representative areas of acquired biopsies. T, tooth; BB, bacterial biofilm; IC, immune cell; AB, alveolar bone. Hematoxylin and eosin (H&E)-stained healthy and periodontitis tissue sections oriented appropriately to resolve the histologic landmarks of interest (bottom). Scale bars, 200 μm. Photomicrographs reflect histologic patterns that were present across all samples (H, *n* = 11; P, *n* = 17 individuals). Panels **a** and **c** created by Ethan Tyler, NIH Medical Arts Branch. **d**, SP and ST segmentation masks of cell borders (SP) and nuclei (ST) colored based on the major cluster annotation (left). Scale bars, 100 μm. Dot plots of major cell population-defining proteins (top) and transcripts (bottom) (right). Expression values are normalized and scaled means. Source data

Oral (gingival) biopsies were acquired from systemically healthy individuals categorized as having either pristine oral health or untreated periodontitis. Tissues were processed for spatial proteomics (SP) using iterative bleaching extends multiplexity (IBEX; Methods) with an 18-parameter antibody panel optimized for oral tissues, and for spatial transcriptomics (ST) using Xenium with a 450-plex panel, including custom probes chosen based on previous single-cell sequencing data^13^. Additional tissues of periodontitis patients were dissociated for single-cell approaches, including CITE-seq^15^ and spectral flow cytometry (Fig. 1a).

Collectively, this newly acquired dataset included over 1 million cells with spatial context, approximately 32,000 cells with paired surface protein and transcriptome data, and over 3.5 million CD45^+^ events from spectral flow cytometry originating from 28 human participants (11 oral health and 17 periodontitis; Fig. 1b and Supplementary Table 1).

Each biopsy was oriented to capture both the external oral epithelium (OE) and internal TAE (Fig. 1c,d). The OE displayed general features of a multilayered squamous epithelium that typically lines the oral mucosa. The TAE was thinner and lacked the typical architectural features of the OE such as keratinization and stratification, but displayed increased spongiosis (edema), indicating fewer inter-epithelial junctions. Notably, a noticeable inflammatory infiltrate was evident in the subepithelial region adjacent to the TAE, even in the setting of clinically diagnosed oral health, suggesting that active homeostatic inflammation is a defining feature of this barrier. In periodontitis, substantial expansion of the inflammatory cell component was documented which extended to distal areas of the subepithelial connective tissue, consistent with the inflammatory nature of the disease (Fig. 1c).

**Extended Data Fig. 1:**
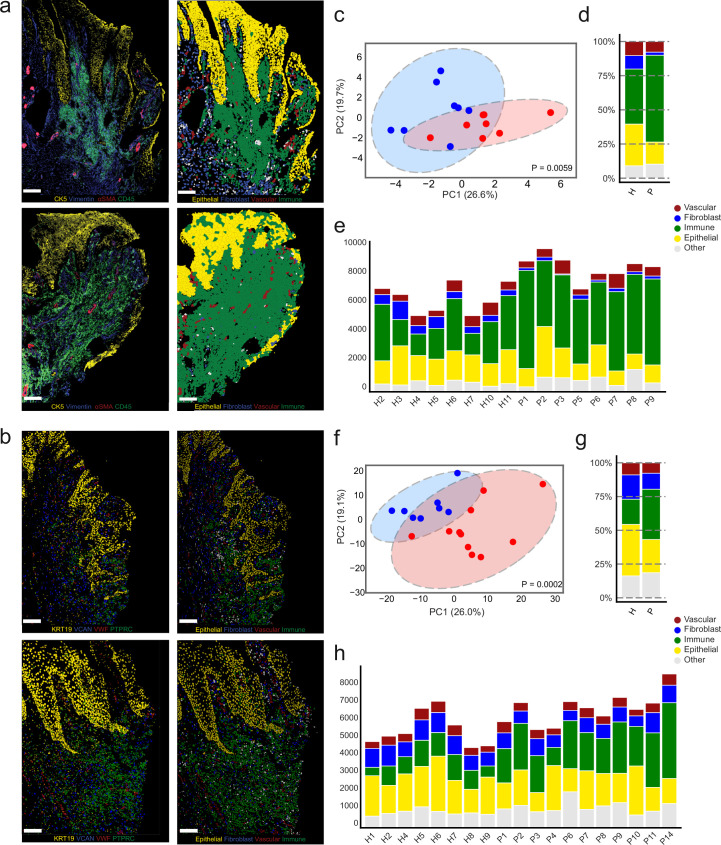
Visualization and quantification of major cellular compartments in human gingiva using IBEX spatial proteomics (SP) and Xenium spatial transcriptomics (ST). **a**) Protein expression and major cell subset annotations from SP in healthy (H) and periodontitis (P) gingival tissue sections shown in Fig. 1d. Scale bars: 100μm. **b**) Transcript expression and major cell subset annotations from ST in H and P gingival tissue sections. Scale bars: 100μm. **C**) Pseudobulk (per-subject) PCA of SP. Points are individual subjects colored by condition (H = blue, P = red); dashed contours show 95% *data* ellipses per group. Separation by status was confirmed by two-sided MANOVA on PC1–PC2 (Pillai’s trace = 0.546, F(2,13) = 7.83, p = 0.0059). **d**) Proportion plots of major cellular compartments in H and P gingival SP sections. **e**) Stacked bar plots showing the absolute number of major compartment cells per mm² of total tissue area in SP samples. **f**) Pseudobulk (per-subject) PCA of ST. Two-sided MANOVA confirmed group separation on PC1–PC2 (Pillai’s trace=0.648, F(2,16) = 14.71, p = 0.0002). **g**) Proportion plots of major cellular compartments in H and P gingival ST sections. **h**) Stacked bar plots showing the absolute number of major compartment cells per mm² of total tissue area in ST samples. Source data

Of note, both spatial proteomic and transcriptomic approaches reliably captured the major cellular compartments of the gingiva (epithelial, fibroblast, endothelial and immune) based on established cell-defining markers (Fig. 1d and Extended Data Fig. 1a,b). Both modalities identified consistent separation between oral health and periodontitis and confirmed known cellular shifts in periodontitis, such as an increase in immune cell numbers at the expense of fibroblasts^13,16^ (Extended Data Fig. 1c–h). Collectively, this dataset provided an anatomically oriented cellular atlas of the human gingiva across several high-throughput modalities in health and inflammatory disease.

### Spatial proteomics and transcriptomics maps cellular distribution

**Fig. 2:**
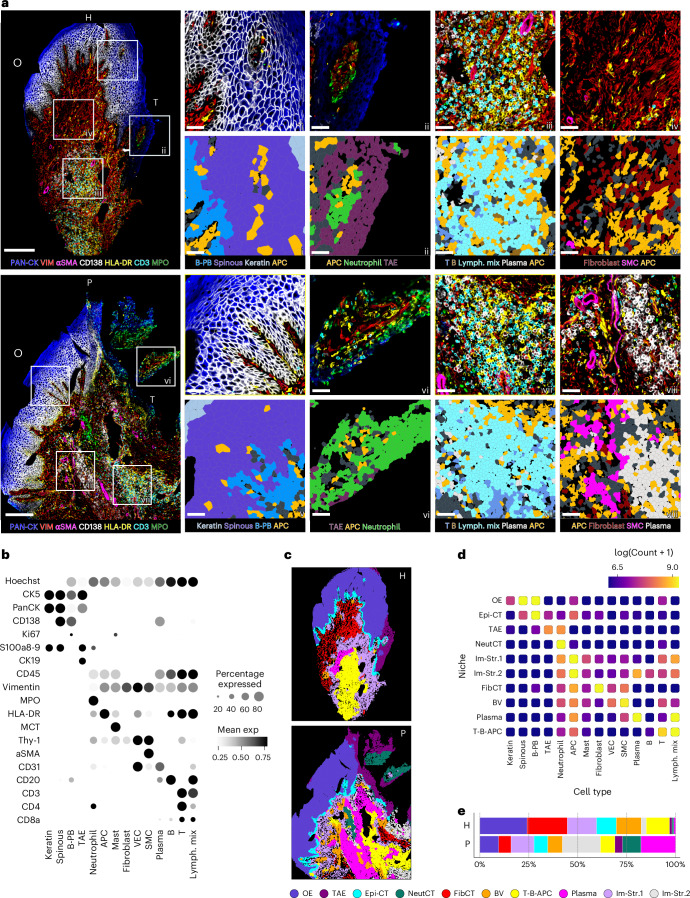
Spatial proteomics (IBEX) resolves major cell subsets in health and disease. **a**, Representative SP images of protein markers defining major cellular compartments in H and P. Insets: cell type annotations with representative immunostaining (top) and segmentation masks (bottom) at the regions of OE (i, v), TAE (ii, vi), subepithelial lymphoid aggregates (iii, vii) and deep connective tissue (iv, viii) in health and disease. Scale bars, 200 μm (large fields of view) and 40 μm (insets). B-PB, basal-parabasal; lymph., lymphoid; SMC, smooth muscle cell. Photomicrographs reflect spatial patterns that were present across all samples (H, *n* = 8, P, *n* = 8 individuals). **b**, Dot plot of protein marker expression across cell subpopulations. Expression values are normalized and scaled means. VEC, vascular endothelial cell; mean exp, mean expression. **c**, Tissue sections depicted in **a**, colored by niche annotation. **d**, Heatmap showing cell type distribution across SP-defined niches. Values represent log-transformed cell counts. Epi-CT, epithelium-connective tissue. **e**, Proportion plots of SP niches in H and P. Source data

**Extended Data Fig. 2:**
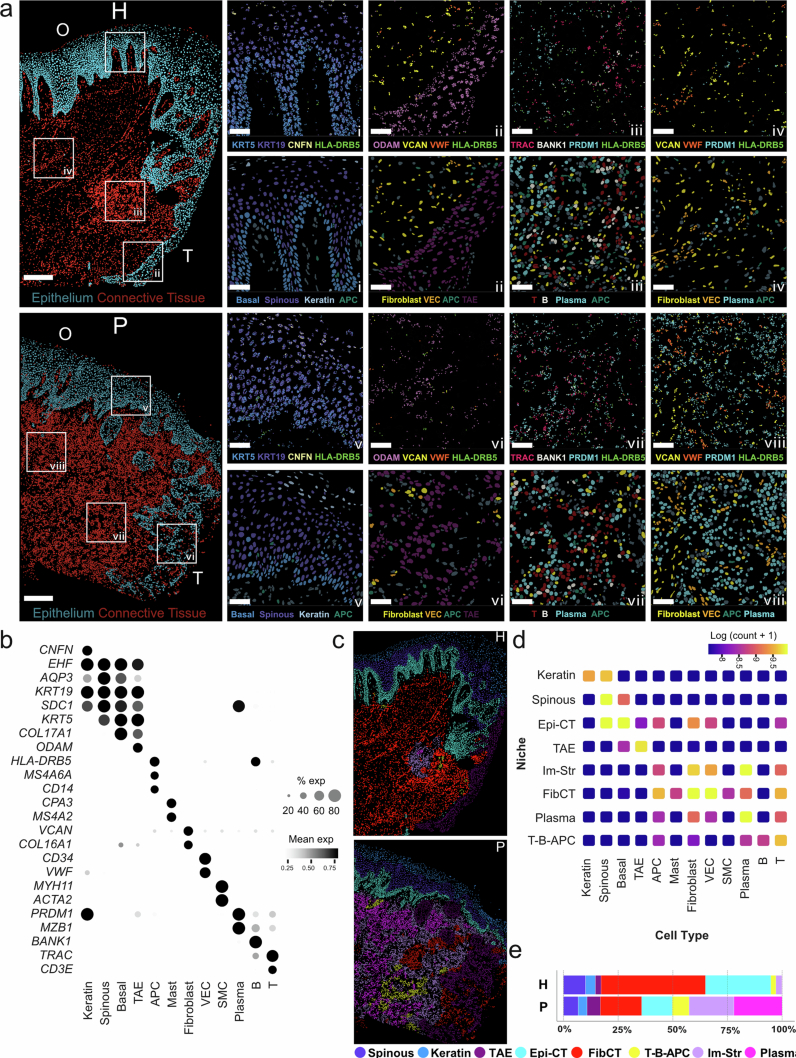
Spatial transcriptomics resolves major cellular compartments in health and disease. **a**) ST nuclei masks of representative H and P tissues colored by general histologic region (epithelial and connective tissue). Insets show transcript localization and cluster annotation for the OE (i,v), TAE (ii,vi), subepithelial area (iii, vii) and deep connective tissue (iv, viii). Scale bars: 200μm (large fields of view) and 40μm (insets). **b**) Dot plot of transcript expression across cell subpopulations. Expression values are normalized and expressed as scaled means. **c**) Tissue sections depicted in (A) colored by the ST niche annotation. **d**) Heatmap showing cell type distribution across ST-defined niches. Values represent log-transformed cell counts. **e**) Proportion plots of ST niches in H and P. Source data

**Extended Data Fig. 3:**
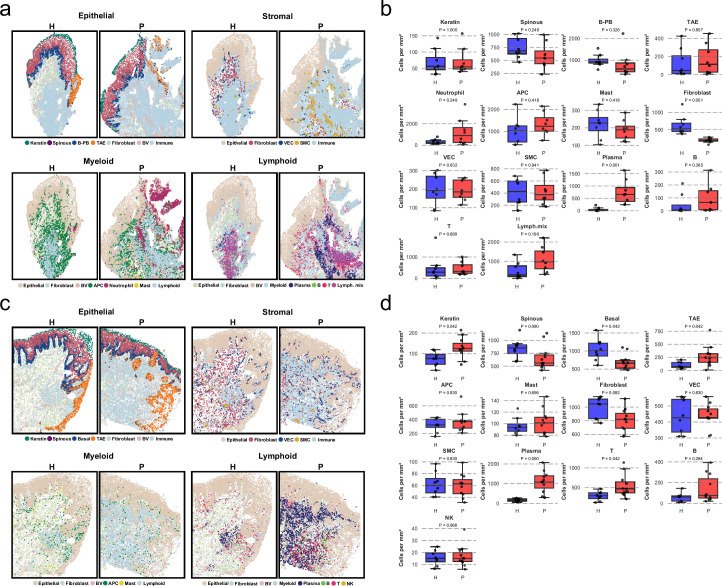
Spatial resolution and quantification of major cell subsets in human gingiva. A) Spatial localization of major SP-defined epithelial, stromal, lymphoid, and myeloid subsets in the same tissue sections as Fig. 2a. **b**) Boxplots showing the number of SP-defined cells per mm² of total tissue area in H and P. Dots represent individual subjects. P values were calculated from two-sided Mann-Whitney U-test after Benjamini–Hochberg FDR correction comparing H (n = 8) versus P (n = 7-8). Boxplot centers indicate median values; box boundaries represent the 25th and 75th percentiles; whiskers extend to the most extreme points within 1.5× the interquartile range (IQR); and minimum/maximum values correspond to the smallest and largest observations within this whisker range. **c**) Spatial localization of major ST-defined epithelial, stromal, lymphoid, and myeloid subsets in the same tissue sections as Extended Data Fig. 2a. **d**) Boxplots showing the number of ST-defined cells per mm² of total tissue area in H and P samples. Dots represent individual subjects. P values from two-sided Mann-Whitney U-test after Benjamini–Hochberg FDR correction comparing H (n = 8) versus P (n = 11) are shown. Boxplot elements follow same conventions as in (B). Source data

Spatial proteomics and spatial transcriptomics were employed in parallel to define cellular compartments and their distribution within healthy and diseased gingiva (Fig. 2 and Extended Data Figs. 2 and 3). IBEX immunostaining enabled identification of major cell subsets within the epithelial, stromal and immune compartments. The OE separated into discrete layers, including basal-parabasal, spinous and keratin based on differential expression of specific markers, including Ki67, CD138 and S100A8/9. The TAE was clearly distinguished from the OE by expression of keratin 19 (CK19) across all layers (Fig. 2a,b). Stromal subpopulations were defined using a combination of classic markers (vimentin, CD31, αSMA and Thy1). Major immune cell subsets were identified based on cell-defining markers and found within the epithelium and subepithelial regions (Fig. 2a,b). HLA-DR^+^ cells were present in the OE and in the subepithelial connective tissue and were defined as B cells, non-B cell antigen-presenting cells (APCs) or as part of a mixed lymphoid population (lymphoid mix). MPO^+^ neutrophils were evident near and within the TAE, with their numbers particularly increased in periodontitis tissues (Extended Data Fig. 3a,b). T (CD3^+^, CD4^+^ or CD8^+^), B (CD20^+^) and APC cell clusters were observed in the subepithelial space in both health and disease. CD45^+^CD138^+^ plasma cell aggregates were evident in periodontitis (Fig. 2a,d and Extended Data Fig. 3a,b).

Discrete tissue neighborhoods were defined using an unbiased niche analysis, reflecting particular cell constituents within specific tissue locations. TAE and OE were defined as distinct epithelial compartments. The stromal compartment consisted of a blood vessel (BV) niche, a fibroblast connective tissue (FibCT) niche and two immune-stromal cell niches containing diverse immune cells (Im-Str.1 and Im-Str.2). Within the subepithelial compartment, distinct immune cell niches included a neutrophil connective tissue niche (NeutCT) adjacent to the TAE, a T cell, B cell, antigen-presenting cell (T-B-APC) niche, and a plasma cell niche. The plasma niche as well as the Im-Str.2 containing plasma cells, were primarily observed in diseased tissues (Fig. 2c–e).

**Extended Data Fig. 4:**
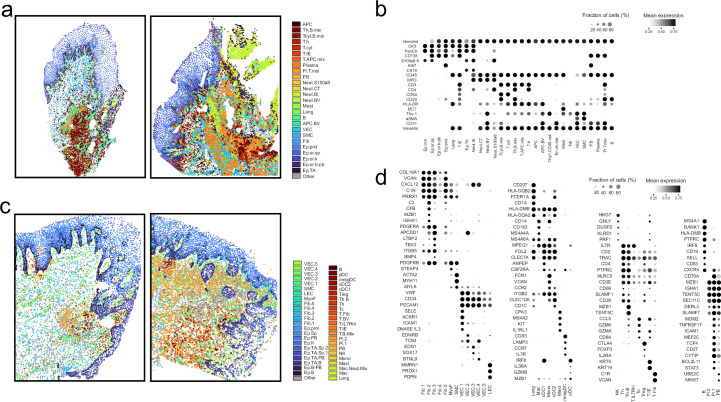
Spatial resolution and marker expression of fine-grained cell subpopulations in human gingiva. **a**) Spatial localization of SP-defined subsets in the same tissue sections as Fig. 2a. **b**) Normalized and scaled protein marker expression per SP-defined subset. **c**) Spatial localization of ST-defined subsets in the same tissue sections as Extended Data Fig. 2a. **d**) Normalized and scaled transcript expression per ST-defined subset. Source data

Spatial transcriptomic analysis confirmed IBEX major cell subsets, their localization and relevant abundance in health and disease (Extended Data Figs. 2 and 3), except for neutrophils, which were sparsely detected with Xenium. However, Xenium allowed for cell subset characterization at a more granular level (Extended Data Fig. 4). The anatomic layers of the OE clustered based on unique gene expression, whereas the TAE clustered separately and expressed signature odontogenic genes such as *ODAM* but lacked keratinization markers such as cornifelin (*CNFN*) (Extended Data Fig. 2a,b).

Major immune cell subsets identified in the subepithelial space included APCs expressing genes encoding *CD14* and *HLA-DRB5*, αβ T (*TRAC*^*+*^, either *CD4*^*+*^ or *CD8*^*+*^) and B cells (*BANK1*^*+*^ and *HLA-DRB5*). Periodontitis-enriched aggregates of plasma cells were defined by *PRDM1* and *MZB1* (Extended Data Fig. 2a,b). Stromal cells were also further detailed based on gene expression defining diverse fibroblast, myofibroblast, smooth muscle, vascular endothelial and lymphatic endothelial cell subsets (Extended Data Fig. 4c,d). Quantification of the spatial transcriptomics subpopulations confirmed a decrease in fibroblasts and increase in plasma cells in the context of periodontitis, consistent with spatial proteomics (Extended Data Fig. 3d).

Niche analysis of the spatial transcriptomics dataset identified epithelial and subepithelial niches consistent with the proteomic approach, albeit with a lack of a neutrophil-containing niches (Extended Data Fig. 2c–e). OE and TAE were resolved as distinct epithelial niches and immune cells were differentially distributed in discrete cell niches, including a T-B-APC niche present in both health and disease and a plasma cell niche and an Im-Str cell niche containing plasma cells primarily observed in periodontitis (Extended Data Fig. 2c–e). In sum, with complementary single-cell resolution approaches we define distinct tissue niche organization in health and disease at the human oral mucosal barrier.

### Neutrophil-dominated inflammation at the TAE

**Fig. 3:**
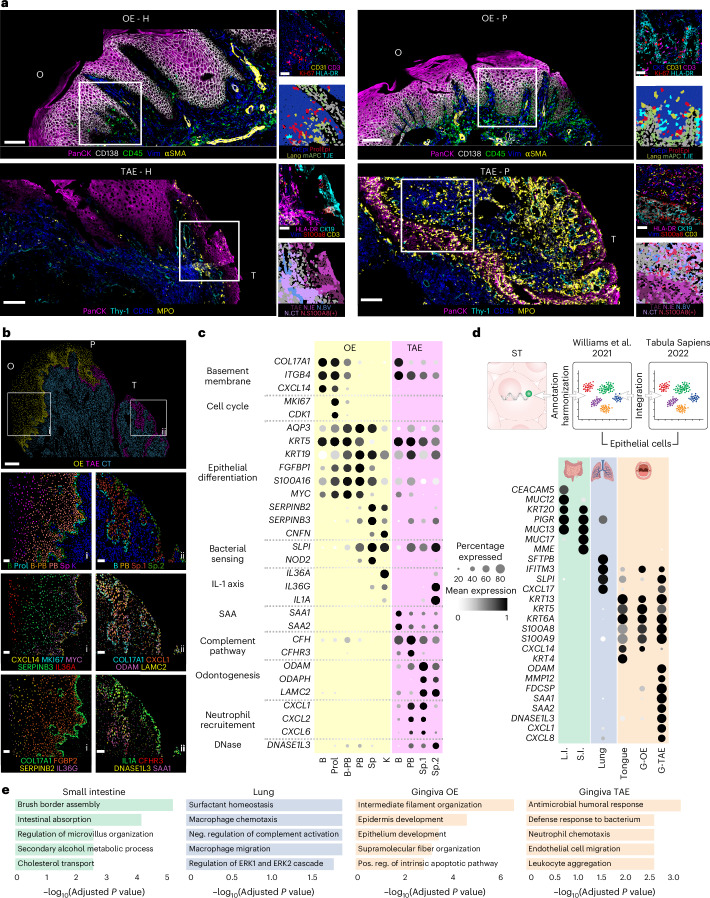
Distinct epithelial compartments within the human gingival barrier. **a**, Select SP marker expression at the OE and TAE in H and P. Insets: fine-level clusters at each site. ProlEpi, proliferating epithelial cells; Lang, Langerhans cells; T.IE, intraepithelial T cells. The neutrophil subsets are intraepithelial (N.IE), blood vessel-associated (N.BV), connective tissue (N.CT) and N.S100a8/9(+). Scale bars, 200 μm (large fields of view) and 40 μm (insets). Photomicrographs reflect spatial patterns that were present across all samples (H, *n* = 8; P, *n* = 8 individuals). **b**, ST nuclei masks of a representative periodontitis section colored by major histologic region. Insets within the OE (i) and TAE (ii) depict epithelial cluster annotations (first row) and select transcript localizations (middle and last row) within these barriers. Scale bars, 200 μm (large fields of view) and 40 μm (insets). **c**, Dot plot showing expression of transcripts associated with unique biological functions in OE and TAE. Expression values are normalized and scaled means. SAA, serum amyloid A; B, basal; Prol, proliferating; PB, parabasal; Sp, spinous; K, keratin. **d**, Dot plot of transcript expression across different epithelial barrier cell subsets following integration of the gingival reference single-cell RNA sequencing dataset^13^ with the Tabula Sapiens reference ^17^. The gingival reference was reannotated after harmonization with ST epithelial annotation. Expression values are normalized and scaled means. L.I., large intestine; S.I., small intestine; G-OE, gingiva-oral epithelium; G-TAE, gingiva-tooth-associated epithelium. Panel **d** (top) created by Ethan Tyler, NIH Medical Arts Branch. Panel **d** (bottom) created in BioRender; Theofilou, V. https://BioRender.com/6vlvhou (2025). **e**, Bar plots displaying the top five Enrichr^51^ Gene Ontology terms for each mucosal epithelial barrier. Source data

**Extended Data Fig. 5:**
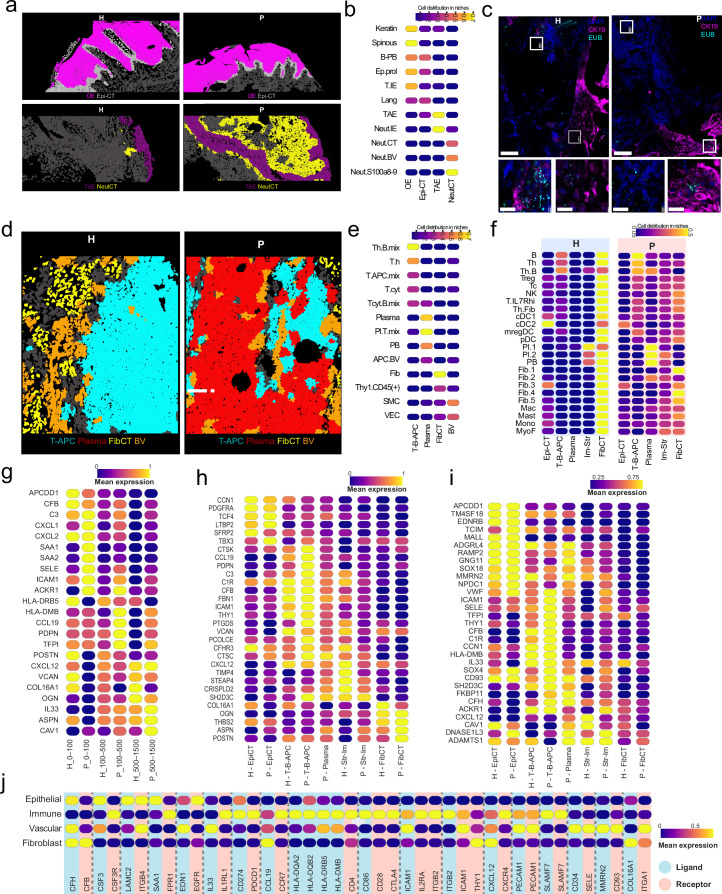
Characterization of epithelial and immune cell niches. **a**) H and P tissue sections, from Fig. 3a annotated by epithelial and subepithelial niche. **b**) Heatmap showing the proportion of specific cell subsets within SP-defined epithelial/subepithelial niches. **c**) Representative FISH images of eubacterial probe (EUB) co-stained with CK19 immunofluorescence in H and P sections. Insets TAE (i) and subepithelial CT regions. Scale bars: 100 μm (main panels), 40 μm (insets). **d**) H and P tissue sections from Fig. 4c and f annotated based on their immune and connective tissue niche. **e**) Heatmap showing the proportion of specific cell subsets within SP-defined niches. **f**) Heatmap showing the proportions of ST-defined cell subsets within immune/connective tissue niches in H and P. G) Heatmap showing relative expression of location-specific genes from Fig. 5c in health (**h**) versus periodontitis (P). Expression values are normalized and scaled means. H) Heatmap showing relative expression of genes in fibroblasts displayed by connective tissue niche in H versus P. Expression values are normalized and scaled means. **i**) Heatmap showing relative expression of genes in vascular cells displayed by connective tissue niche in H versus P. Expression values are normalized and scaled means. **j**) Heatmap showing relative expression of ligand–receptor pairs from Fig. 5g across major cellular compartments. Expression values are normalized and scaled means. Source data

The unique histological characteristics and inflammatory infiltrate at the TAE niche compared to the OE prompted us to further characterize these distinct epithelial regions of the oral mucosa. Besides its multilayered stratification and keratinization, the OE expressed CD138, a protein involved in cell–cell adhesion between squamous epithelial cells, suggesting epithelial integrity (Fig. 3a). Within the OE, scattered immune cells were present, including intraepithelial CD3^+^ T cells (T.IE) and CD45^+^/HLA-DR^+^ cells presumed to be Langerhans cells (Fig. 3a and Extended Data Fig. 5a,b), in both health and disease.

In contrast to the OE, the TAE expressed CK19 and lacked CD138 expression, indicating decreased barrier integrity and increased permeability (Fig. 3a). Consistent with increased permeability, the TAE displayed substantial infiltration by neutrophils. In both health and disease, neutrophils expressing MPO with/without S100A8/9 were identified within the TAE, in proximity to the regional blood vessels, and within the connective tissue. Subepithelial neutrophils were markedly increased in disease, forming a distinctive border immediately beneath the TAE (Fig. 3a and Extended Data Fig. 5a,b). Given the proximity of this highly permeable barrier with the tooth-associated microbial biofilm, we investigated microbial translocation within and beyond the TAE (Extended Data Fig. 5c). Using 16S fluorescence in situ hybridization (FISH) we visualized penetration of microbiota within the CK19^+^ TAE and in the subepithelial space in both health and disease (Extended Data Fig. 5c). Our work highlights the TAE as a highly exposed and penetrable barrier patrolled by infiltrating neutrophils in health, which massively increase in number in the setting of periodontitis.

### A unique immune signature at the TAE

The presence of a localized immune infiltrate at the TAE barrier prompted us to investigate whether epithelial-intrinsic factors participate in the local organization of immunity. Indeed, analysis of our epithelial-specific spatial transcriptomics dataset revealed unique signatures at the OE versus the TAE (Fig. 3b,c). In the OE, epithelial layers displayed distinct gene expression profiles. Basal OE expressed *CXCL14* (Fig. 3b,c), whereas parabasal layer, spinous layer and keratin layer cells were similarly separated by their defining genes (Fig. 3b,c). Generally, the basal and parabasal layers expressed progenitor (*KRT5*), proliferation (*MKI67* and *CDK1*) and hemidesmosomal organization markers (*COL17A1* and *ITGB4*) whereas the spinous and keratin layers expressed genes encoding microbial sensing and immune responsiveness (*NOD2*, *IL36A* and *SLPI*) (Fig. 3b,c).

The transcriptomic profile of the TAE suggested a unique functionality for this barrier (Fig. 3b,c). In addition to genes of odontogenic origin (*ODAM* and *ODAPH*), TAE cells strongly expressed genes encoding neutrophil chemoattractants (*CXCL1*, *CXCL2* and *CXCL6*), suggesting a functional role for the TAE in neutrophil recruitment. The TAE also broadly expressed other immune-related genes, including complement genes (*CFH* and *CFHR3*), and genes encoding acute-phase reactants (*SAA1* and *SAA2)* and the inflammation regulator *DNASE1L3*. Consistent with its anatomy, the outer layers of the TAE (Sp.1/2) lacked gene expression related to keratinization (for example *CNFN*) but instead expressed *LAMC2* which enables hemidesmosomal attachment of TAE to the tooth surface. The superficial layers of the TAE uniquely expressed high levels of *IL1A* and *IL36G*.

To determine if the inflammatory signature of the TAE is unique, we compared the epithelial gene expression of the gingiva with that of other barrier tissues under homeostatic conditions. We cross-referenced our spatial transcriptomics epithelial cell annotations to the epithelial cells in our previously published oral mucosa single-cell dataset^13^, and integrated them with healthy skin, lung and intestinal epithelial cells from the Tabula Sapiens atlas^17^. The oral epithelia (TAE, OE and tongue) displayed a unique transcriptomic signature compared to non-oral epithelial barriers. Notably, the TAE uniquely expressed signature genes encoding acute-phase proteins (*SAA1/2*), neutrophil chemokines (*CXCL1/8*) and *DNASE1L3*, whereas other genes such as *S100A8/9* were shared with other oral compartments (Fig. 3d). Pathway analysis based on top differentially expressed genes across all evaluated compartments revealed classic ontologies upregulated in relevant organs, including ‘intestinal absorption’ in the small intestine, ‘surfactant homeostasis’ in the lung and ‘epithelial development’ in the OE. In contrast, the TAE was defined by a state of inflammatory immune responsiveness driven by constitutive expression of genes associated with inflammatory pathways such as ‘antimicrobial response’, ‘neutrophil chemotaxis’ and ‘leukocyte aggregation’, reflecting a unique mucosal environment poised to rapidly respond to local insults.

### Unique immune zonation at the TAE

**Fig. 4:**
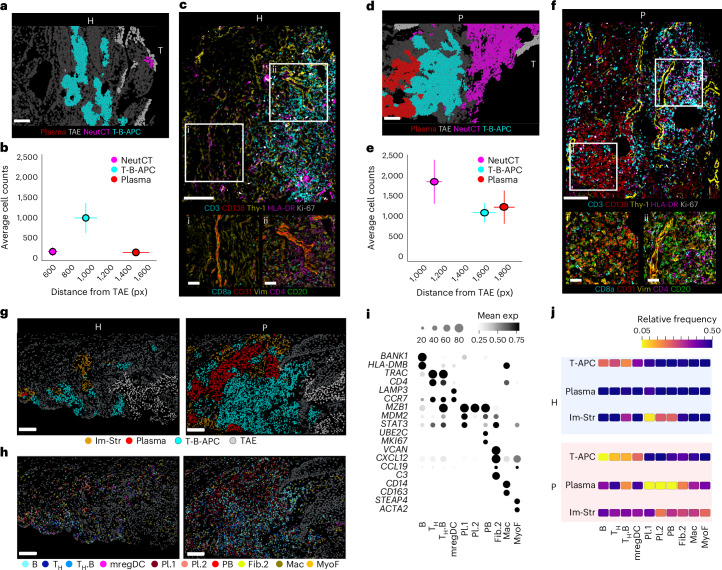
Immune zonation and characterization of lymphoid cell aggregates. **a**,**d**, Representative H (**a**) and P (**d**) sections showing SP-defined immune cell niches and TAE. Scale bars, 100 μm. **b**,**e**, Distance measurement of the SP immune cell niches from the TAE plotted against the number of cells in each niche in H (*n* = 4 or 5 sections from 4 or 5 individuals, **b**) and P (*n* = 9 or 10 sections from 6 or 7 individuals, **e**) sections. Data represent mean values and error bars indicate s.e.m. **c**,**f**, Representative SP images of protein marker expression at subepithelial and the deep connective tissue in health and periodontitis. Scale bars, 100 μm (large field of view) and 30 μm (insets). Photomicrographs reflect spatial patterns that were present across all samples (H, *n* = 8 (**c**); P, *n* = 8 (**f**) individuals). **g**, ST-defined immune cell niches in relation to the TAE in H and P. Scale bars, 100 μm. **h**, Tissue sections depicted in **g**, colored by cell type annotation. Scale bars, 100 μm. **i**, Dot plot showing transcript expression across the cell subsets depicted in **g** and **h**. Expression values are normalized and scaled means. **j**, Heatmap showing the proportion of specific cell subsets within ST-defined immune niches in H and P. Source data

We next aimed to further define the spatial organization of immune cell niches adjacent to the TAE using spatial proteomics. Calculating the average distance of immune-specific niches (NeutCT, T-B-APC and plasma) from the TAE, identified a consistent immune zonation, largely preserved in both health and disease (Fig. 4a,b,d,e). NeutCT was located closest to the TAE, followed by the T-B-APC zone, whereas the plasma niche was localized deepest within the subepithelial tissue. The zonation of NeutCT and T-B-APC was notably consistent between health and disease. In disease, neutrophil numbers substantially increased and extended within deeper areas of the tissue. The T-B-APC zone consisting mainly of helper T (T_H_) (CD4^+^), T_H_-B (CD3/CD4/CD20) and T-APC (CD3/HLA-DR) cells had similar cell numbers in health and disease but extended to deeper areas within the disease tissues (Fig. 4 and Extended Data Fig. 5d,e). In health, distal to the T-B-APC zone, fibro-vascular connective tissue was evident and only contained scattered immune cells (Fig. 4c). Finally, the plasma zone (consisting mainly of CD138^+^ plasma cells, along with T cells and scattered Ki67^+^ plasmablasts) was only evident in disease at areas distal to the T-B-APC zone (Fig. 4 and Extended Data Fig. 5d,e).

Spatial transcriptomics recapitulated the distinctive immune zonation neighboring the TAE (Fig. 4g) and provided a more granular cell subset characterization for each zone (Fig. 4h–j and Extended Data Fig. 5f). The T-B-APC zone was enriched in B cells (*MS4A1/BANK1/HLA-DMB*^*+*^) and T_H_ (*CD2/TRAC/CD4*^+^) cells and contained *CD83/LAMP3/CCR7*^+^ dendritic cells (mregDCs) and a subset of T_H_ cells in proximity to B cells (T_H_.B) in both health and disease. The plasma cell niche was mostly evident in disease and contained distinct subsets of plasma cells (Pl.1 and Pl.2) as well as plasmablasts, the T_H_.B cell subset as well as a specific subpopulation of fibroblasts (Fib.2) expressing *CXCL12*, CCL19 and complement genes (*C3* and *CFB*) (Fig. 4i,j and Extended Data Fig. 5f). In health, relevant cell types were less likely to reside within the plasma cell niche but rather were scattered within the fibrous connective tissue (Im-Str and Fib.CT). Surrounding the immune cell niches an immune-stromal niche (Im-Str) contained scattered immune cells within the stroma (Fig. 4g–j and Extended Data Fig. 5f).

Collectively, these data reveal unique zonation of inflammation at the tooth-associated barrier preserved in health and disease, with increased complexity and organization of lymphoid aggregates in the setting of inflammatory pathology.

### Epithelial and stromal compartments coordinate immune zonation

**Fig. 5:**
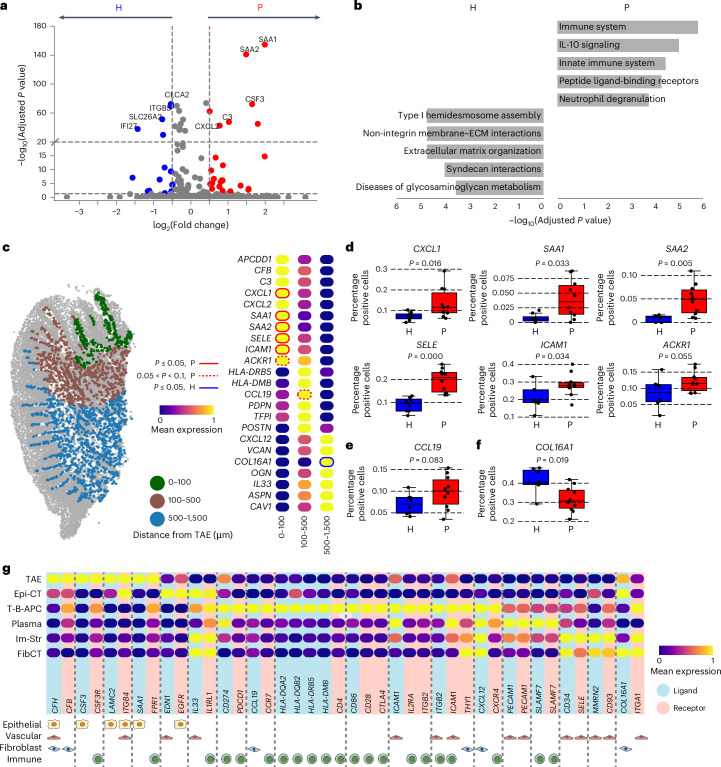
Epithelial and stromal support of immune zonation at the oral barrier. **a**, Volcano plot showing top transcripts enriched in H (blue) versus P (red) TAE. Differential gene expression was assessed using a two-sided Wilcoxon rank-sum test. *P* values were corrected with the Benjamini–Hochberg false discovery rate. **b**, Bar plots depicting the top five Enrichr^51^ Reactome Pathways 2024 enriched in H versus P TAE. The pathways were derived from the top 20 differentially expressed genes per group obtained using a two-sided Wilcoxon rank-sum test. **c**, Stromal (fibroblast and vascular) cells colored by distance from the TAE in a representative periodontitis section (left). Heatmap of genes expressed by stromal cells across distance-defined areas (right). Outlines indicate transcripts with significantly (*P* ≤ 0.05) higher proportions of stromal cells expressing them in P (red) or H (blue), as determined in **d**–**f**. Dotted outlines mark trends toward significance (0.05 < *P* < 0.1). Expression values are normalized and scaled means. **d**–**f**, Boxplots showing the proportion of stromal cells expressing the transcripts identified in **c** within the 0–100 μm (**d**), 100–500 μm (**e**) and 500–1,500 μm (**f**) areas. Dots represent individuals. *P* values from a two-sided Mann–Whitney *U*-test of H (*n* = 7 or 8) versus P (*n* = 10) are indicated. Boxplot centers indicate median values; box boundaries represent the 25th and 75th percentiles; whiskers extend to the most extreme points within 1.5× interquartile range (IQR); and minimum/maximum values correspond to the smallest and largest observations within this whisker range. **g**, Heatmap of gene expression for ligand–receptor pairs from the OmniPath reference list^18^ coexpressed within the same niches. Predominant cell type expressing each ligand or receptor, as determined in Extended Data Fig. 5j (bottom). Expression values are normalized and scaled means. Images in **g** created in BioRender; Theofilou, V. https://BioRender.com/trpkfmz (2025). Source data

The unique immune zonation adjacent to the TAE prompted us to examine whether nonimmune/structural cells participate in the organization of immunity in a location-specific manner. We first inquired about the inferred functionality of the TAE itself in health and periodontitis. TAE in disease displayed a significant upregulation of genes encoding innate immune regulators (*SAA1/2* and *C3*) and neutrophil chemoattractants (such as *CXCL2* and *CSF3*), consistent with increased accumulation of neutrophils in the setting of inflammatory disease (Fig. 5a). Pathway enrichment further underscored these functions revealing an enrichment in immune-related pathways, including ‘innate immune system’ and ‘neutrophil degranulation’ in periodontitis (Fig. 5b).

We next examined possible stromal cell contributions in the organization of location-specific immunity, taking a niche-agnostic approach to investigate stromal transcriptomes as a function of distance from the TAE (0–100 µm, 100–500 µm and 500–1,500 µm). Top genes expressed within the stromal compartment revealed unique location-specific gene expression patterns (Fig. 5c). As such, stromal cells proximal to the TAE (0–100 µm) expressed genes encoding immune cell adhesion molecules (*SELE* and *ICAM1*), neutrophil chemoattractants (*CXCL1* and *CXCL2*), and acute-phase reactants (*SAA1* and *SAA2*), mirroring TAE expression patterns and supporting a potential role for the stromal compartment in the coordination of innate immunity at this interphase. Intermediate distance (100–500 µm) stromal cells expressed genes linked to antigen presentation (*HLA-DRB5* and *HLA-DMB*) and tertiary lymphoid structure (TLS) organization (*CCL19)*, consistent with the presence of lymphoid aggregates. Distal stromal cells (500–1,500 µm) also displayed a unique signature with high expression of the gene encoding an immune cell chemoattractant *CXCL12* (Fig. 5c). Of note, some of the area-specific genes were differentially expressed between health and disease. Genes encoding innate molecules, neutrophil chemoattractants (*SAAs* and *CXCL1*) and endothelial adhesion molecules (*SELE* and *ICAM1*) were upregulated in disease in the area proximal to the TAE, chemokine *CCL19* was upregulated in disease in the intermediate distance area, whereas the classic structural gene (*COL16A1*) was significantly upregulated in the distal area in health. Beyond global stromal transcriptomic signatures, we further interrogated fibroblast and vascular cell gene expression patterns across our previously defined niches to gain an overview of cell-specific inferred functionality in health and disease (Fig. 5d–f and Extended Data Fig. 5g–i).

Finally, we analyzed our spatial transcriptomics dataset for potential ligand–receptor interactions using the OmniPath database^18^, enabling identification and visualization of both nonimmune–immune and immune–immune pairings within each previously defined niche (Fig. 5g and Extended Data Fig. 5j). The TAE niche showed coordinated expression of genes encoding neutrophil-associated ligand–receptor pairs (*CSF3–CSF3R* and *SAA1–FPR1*). Within the T-B–APC niche, expression of genes encoding ligand–receptor pairs implicated in TLS development were observed, including *CD274–PDCD1*, *CCL19–CCR7* and *CXCL12–CXCR4*, and antigen presentation and costimulation molecule pairs (*HLA-DR-DM/CD4* and *CD28/CD80/CTLA4*). Finally, the plasma cell niche displayed distinct coordinated expression of a gene encoding a plasma cell signaling molecule (*SLAMF7*) (Fig. 5g).

Collectively, these data suggest location-specific roles for both the epithelial and stromal compartments in the organization of immunity into zones at the tooth-associated mucosal barrier.

### Tertiary lymphoid structures form in periodontitis

**Fig. 6:**
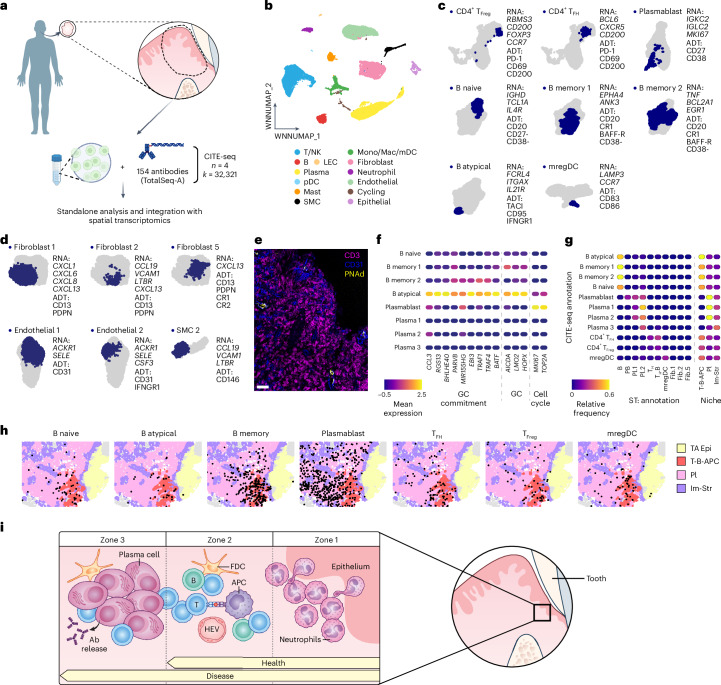
Immature tertiary lymphoid structures in periodontitis tissues. **a**, P tissue samples were processed for protein-full transcriptome (CITE-seq) analysis. CITE-seq datasets integrated with ST to provide inferred spatial context. Images in **a** created in BioRender; Theofilou, V. https://BioRender.com/5icfcmc (2025). **b**, Weighted nearest neighbor (WNN) uniform manifold approximation and projection (UMAP) representation of the major cellular compartments identified via CITE-seq. **c**,**d**, UMAP (left) and defining RNA transcripts and antibody-derived tags (ADTs, right) for selected CITE-seq immune (**c**) and stromal (**d**) cell clusters. **e**, Immunofluorescence staining for CD3, CD31 and PNAd in a representative P tissue section. Scale bar, 40 μm. Immunofluorescence staining pattern was representative of five periodontitis samples. **f**, Heatmap showing relative expression of genes associated with GC commitment, GC and class switching, and cell cycle across CITE-seq B and plasma cell subsets. **g**, Heatmaps displaying concordance between selected ST and imputed CITE-seq annotations (left) and the relative abundance of imputed CITE-seq clusters within ST-defined immune niches (right) following integration of the two approaches. **h**, Spatial location of imputed CITE-seq cluster annotations superimposed on ST-defined immune niches and TAE in a representative P section. **i**, Schematic summary of the key findings of this study Panel **i** created by Ethan Tyler, NIH Medical Arts Branch. Source data

**Extended Data Fig. 6:**
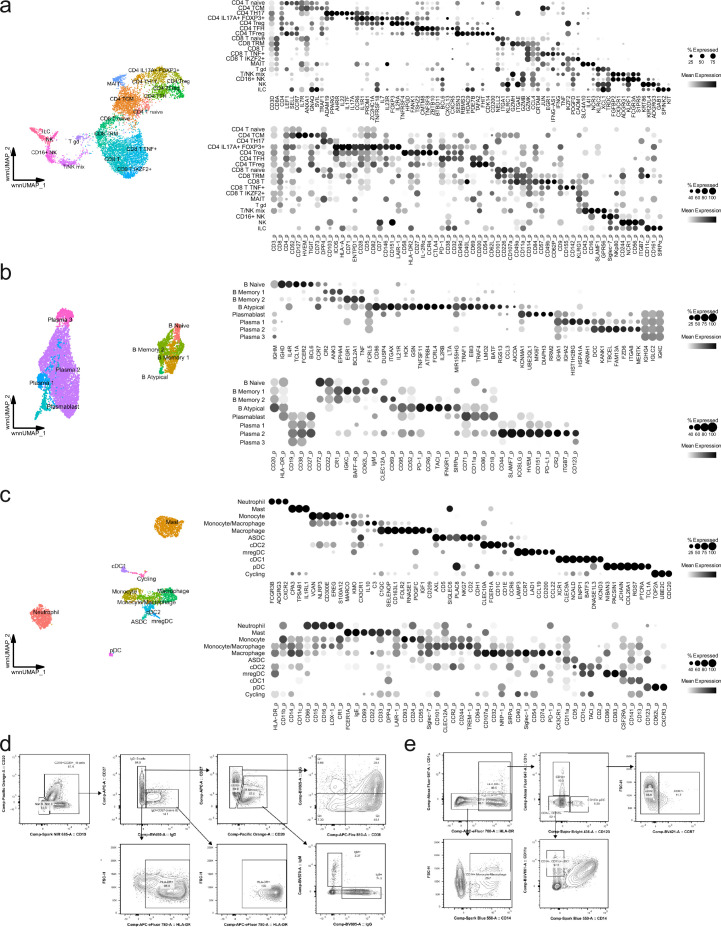
Detailed characterization of immune cell subsets in P tissues by CITE-seq and spectral flow cytometry. **a**) UMAP (left), defining transcripts (upper right) and defining ADTs (lower right) of CITE-seq – defined T, NK, and ILC subpopulations. **b**) UMAP (left), defining transcripts (upper right) and defining ADTs (lower right) of CITE-seq - defined B and Plasma cell subpopulations. **c**) UMAP (left), defining transcripts (upper right) and defining ADTs (lower right) of CITE-seq - defined myeloid and pDC subpopulations. **d**) Representative spectral flow cytometry plots of Live, CD45 + , SSC low, CD3– B and plasma cells including CD27–, IgD + , HLA-DR+ naïve B cells, CD20 + , CD27–, IgM+ or IgG+ B cells, and CD20–, CD27 + , CD38 + , IgG +/– plasma cells. **e**) Representative spectral flow cytometry plots of Live, CD45 + , CD3–, CD19–, CD66b– myeloid populations including CD1C–, CD14+ monocyte-macrophages, HLA-DR + , CD1C + , CCR7– cDC2s, HLA-DR + , CD1C + , CCR7+ mreg DCs, HLA-DR + , CD123+ pDCs, HLA-DR + , and CD1C–, CD123–, CD14–, CD11C + cDC1s. Source data

**Extended Data Fig. 7:**
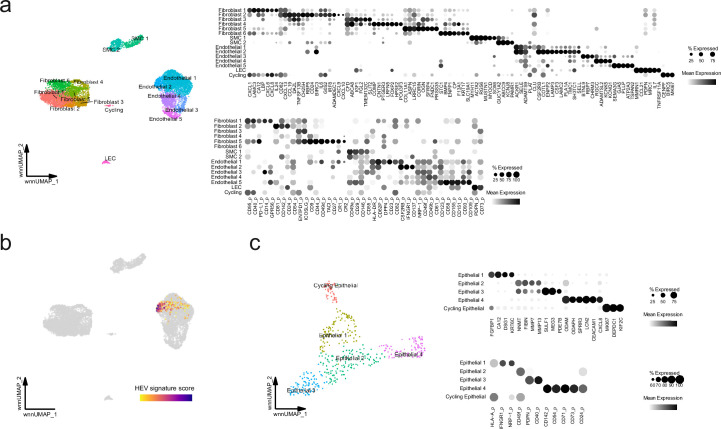
CITE-seq characterization of nonimmune cell subsets in P tissues. **a**) UMAP (left), defining transcripts (upper right) and defining ADTs (lower right) of CITE-seq – defined stromal subpopulations. **b**) UMAP of stromal subpopulations colored based on the HEV-signature score. **c**) UMAP (left), defining transcripts (upper right) and defining ADTs (lower right) of CITE-seq - defined epithelial cell subpopulations. Source data

The presence of organized lymphoid aggregates with distinct zonation suggested that TLSs may form proximal to the TAE in the context of periodontitis. To determine whether TLS-associated cell types are present and to further delineate the inflammatory compartment in periodontitis, we employed CITE-seq^15^ to simultaneously capture epitopes and transcriptomic signatures at a single-cell level (Fig. 6a). Analysis of >32,000 cells from dissociated periodontitis-affected gingiva allowed for in-depth characterization of the immune and stromal cell compartments with increased confidence compared to our previous single-cell RNA sequencing studies^13^ (Fig. 6b and Extended Data Figs. 6 and 7).

TLS-related cell types within the T cell compartment in periodontitis (Extended Data Fig. 6a) included follicular helper T (T_FH_) cells (CD4^+^ T_FH_; *PRDM1*^*−*^, *BCL6*^*+*^, *CXCR5*^*+*^ and *CD200*^*+*^) and follicular regulatory T (T_Freg_) cells (CD4^+^ T_Freg_; *PRDM1*^*−*^, *RBMS3*^*+*^, *CD200*^*+*^ and *FOXP3*^*+*^)^19^ (Fig. 6c). Both T_FH_ and T_Freg_ populations expressed PD-1, CD69 and CD200 epitopes. The full spectrum of B cell maturation was also annotated, encompassing naive B cells (*IGHD*^*+*^, *TCL1A*^+^, *IL4R*^*+*^, CD20^+^, CD27^*−*^ and CD38^*−*^), two populations of memory B cells (CD20^+^, CR1^+^, BAFF-R^+^ and CD38^−^) as well as atypical B cells (*FCRL4*^*+*^, *ITGAX*^*+*^, *IL21R*^*+*^, TACI^+^, CD95^+^ and IFNGR1^+^) (Fig. 6c). The B atypical cluster contained cells with markers indicative of germinal center (GC) commitment, GC identity (*EBI3* and *TRAF1*) and class switch recombination (*AICDA*)^19,20^ (Fig. 6f). Plasma cells were clearly defined and included a portion of proliferating (*MKI67*^+^*TOPA2*^*+*^) plasmablasts (Fig. 6c and Extended Data Fig. 6b). Using spectral flow cytometry, we further confirmed the presence of naive/memory B cells and plasma cells. The majority of B cells were HLA-DR^+^ and 40–70% of plasma cells and memory B cells were IgG^+^. Within the APC compartment, we confirmed the presence of mregDCs (*CCR7*^*+*^, *LAMP3*^*+*^, CD83^*+*^ and CD86^*+*^) and further defined dendritic cell populations (Fig. 6c and Extended Data Fig. 6c–e).

This approach additionally identified stromal cell subsets with the potential to support the TLS environment. Three subsets of fibroblasts expressed features defining fibroblast reticular cells, including *CXCL13* and PDPN. Fibroblast 2 and smooth muscle cell 2 (SMC2) further expressed *CCL19*, *VCAM1* and *LTBR*, suggesting that T-zone reticular- and perivascular reticular-like cell populations reside within the gingiva^21^ (Fig. 6d). We further identified a vascular endothelial subset distinguished by *CSF3* and IFNGR1 expression and enriched in a gene signature associated with high endothelial venules (HEVs)^22^ (Fig. 6d and Extended Data Fig. 7b). The presence of HEVs was further confirmed by peripheral node addressin (PNAd) staining within lymphoid aggregates in periodontitis tissues (Fig. 6e).

To predict the spatial context for key TLS-related cell subsets in periodontitis identified in the CITE-seq data, we integrated the spatial and CITE-seq transcriptomic datasets. B cell populations and mregDCs showed high levels of alignment between approaches, whereas the CITE-seq defined CD4^+^ T_FH_ and CD4^+^ T_Freg_ clusters had the highest similarity with the T_H_ cells in proximity to B cell (T_H_.B) cluster identified with spatial transcriptomics (Fig. 6g). Spatial mapping of CITE-seq clusters demonstrated enrichment of TLS-related B cell, T cell and mregDC clusters within the T-B-APC niche, and plasmablasts within the plasma (Pl) and immune-stromal (Im-Str) niches (Fig. 6g–h).

Collectively, through an integrated multiomics approach, we identify unique immune zonation adjacent to the TAE preserved in health and disease, with formation of immature TLS structures (Fig. 6i) with the potential to support local antibody production in inflammatory disease.

## Discussion

Our work reveals insights into tissue-specific wiring of immunity at the TAE, a human mucosal barrier that is not only exposed to the outside environment but is endowed with an inherent ability for infection protection and for optimal wound healing despite constant injury. We thus deduce that lessons learned from this ‘healing-privileged’ mucosal site, are not only of interest toward understanding tissue-specific immunity but could provide insights that can conceivably be therapeutically leveraged for optimal barrier immune responsiveness at other tissues.

The TAE is indeed a particularly permeable and exposed barrier compartment within the oral cavity^23^. Within this specialized local epithelial niche, we uncover a unique transcriptome dominated by neutrophil chemoattractants and antimicrobial, acute phase and damage-related inflammatory proteins. Such an inflammatory signature is not typically encountered in healthy epithelia. Instead, it resembles the epithelial responses observed in inflammatory diseases such as psoriasis^24^ and IBD^25^ in skin and the gastrointestinal tract; however, how the local epithelial niche is conditioned toward inflammatory responsiveness is not completely understood^26,27^. It is conceivable that both the cell-intrinsic wiring of this odontogenic-origin barrier and/or local microbial triggers promote inflammatory responsiveness.

Consistent with the epithelial transcriptome, there is specific infiltration of neutrophils within the inter-epithelial junctions at this interface and in the immediate subepithelial space in both health and disease, with marked expansion in disease. Neutrophils are evident in regional vessels, transmigrating from the vascular space within the tissue and found through the intraepithelial junctions of the permeable TAE. The phenomenon of neutrophils exiting the healthy TAE into the oral cavity has long been recognized as a unique feature of the oral barrier^28,29^. In contrast to other barriers where neutrophil transmigration indicates infection or inflammation, neutrophils in the oral mucosa are broadly recognized to have homeostatic functions as patients with defects in neutrophil development and/or trafficking are highly susceptible to severe oral inflammation and periodontitis^30,31^. While health-associated neutrophils are clearly linked to barrier homeostasis, neutrophil expansion is associated with disease immunopathology in periodontitis, highlighting the importance of balanced neutrophil responses within this environment^32–34^. Whether specific neutrophil subsets^28,35^, location-specific responses or divergent tissue cues in health and disease are linked to the protective and pathogenic roles of neutrophils at this barrier remains largely unexplored and intriguing.

Beneath the immune-active epithelial-neutrophil zone, we document areas of adaptive immune activity formed by T, B and APC cells in both health and disease. It is conceivable that local APCs sample the microbiota and other antigens, present them locally to T cells, and induce T cell-specific immunity to local antigens. Of note, this distinctive immune zonation formed by epithelial/neutrophil border with an underlying lymphocyte–APC zone is preserved in both health and disease, suggesting preserved coordination of immune responsiveness at this barrier.

In untreated inflammatory disease, this distinct inflammatory compartmentalization is maintained with the addition of a zone of plasma cells intermixed with lymphoid aggregates further organized into structures indicative of immature TLSs. While these structures lack distinct T, B and GC zones, they contain necessary cellular components to generate a local adaptive immune response and local antibody production. Key cellular subsets defined include CD4^+^ T_FH_ and T_Freg_ cells, atypical/GC-like B cells, mregDC, plasma cells and plasmablasts, consistent with findings in other chronic inflammatory diseases^36^. Building upon findings from our previous single-cell oral mucosal atlas, which identified fibroblast subpopulations with immune functionality^13^, we describe stromal cells with location-specific transcriptomes suggesting their potential to support immune zonation and TLS formation. As such stromal cells (particularly fibroblasts) in the area just beneath the TAE express innate immune molecules and neutrophil chemoattractants related to the recruitment of neutrophils from the vasculature into the barrier. In deeper areas stromal cells (both fibroblasts and endothelial cells) express molecules related to the recruitment, retention and activation of immune cells, with increased expression of relevant ligand-pairs in the setting of inflammatory disease. Of interest, particularly fibroblasts within the T-B-APC niche expressed molecules associated with the development of TLSs, such as fibroblasts expressing *CXCL13*, *CCL19*, *VCAM1* and *LTBR*^37^. How health-associated T, B and APC clusters transform into TLS during the progression from health to disease cannot be dissected in a human study; however, stromal cell populations acting as lymphoid tissue organizer (LTo) cells may facilitate the transition from healthy to diseased oral mucosa. These LTo-like cells may integrate inflammatory signals to recruit and retain immune cells in subepithelial niches which eventually form immature TLSs and promote the conversion of T cells into T_FH_ cells^38,39^, ultimately supporting local antibody production. Indeed, the local presence of B cells with features of class switching^40^ combined with plasma and plasmablast aggregation, strongly suggests local antibody production occurs in the gingival mucosa, particularly in periodontitis. What remains unknown is both the specificity of these antibodies and the functionality of local antibody responses in both health and disease. Given the constant microbial stimulation and tissue injury at the gingiva, we speculate that antibody responses here are largely commensal-specific, serving to survey local microbiota^39^ and protect against invasive infection. Immune spatial organization has been shown to be more efficient than uniform distribution of immune cells in protection against bacterial dissemination in other organs such as the liver^41^. This process becomes increasingly important in the context of periodontitis, where the microbiome load is significantly higher^42^, and microbial translocation is increased^43^ conceivably participating in systemic disease triggering^44,45^. Whether this theoretically protective antibody response can contribute to local tissue destruction^46^ in periodontitis remains unclear. Moreover, while adaptive T cell-mediated immunity is strongly linked to inflammatory disease pathology in experimental periodontitis^47–50^, it is crucial to determine whether a TLS-mediated adaptive immune response, including local antibody production, protects the oral mucosal barrier or contributes to mucosal tissue destruction and bone resorption in periodontitis.

Dissecting the mechanisms that promote host–environment symbiosis versus inflammation at this barrier is critical toward preventing and/or treating the prevalent human inflammatory disease periodontitis and potentially circumventing its complex disease comorbidities^45^. Furthermore, understanding how tissue-specific immune responsiveness protects from invasive infection protection and promotes optimal wound healing can provide insights that can be leveraged for therapeutic interventions in diverse disease states.

## Methods

### Human participant and clinical study details

#### Human volunteer recruitment and ethics approval

Volunteers were prospectively recruited under an Institutional Review Board-approved clinical protocol at the National Institutes of Health (NIH) Clinical Center (ClinicalTrials.gov Identifier NCT01568697). Participants provided written consent for inclusion in the study. Participants were reimbursed for the inconvenience of study visits. All human samples and data were collected within the NIH Clinical Center and the NIH Intramural Program. Samples and data were collected for this study between 2021 and 2024.

All enrolled participants were systemically healthy adults, screened for lack of systemic comorbidities, excluding individuals with active malignancy, history of radiation to the head and neck region, chemotherapy or radiation within the last 5 years, history of hepatitis B/C or HIV infection, autoimmune diseases and diagnosis of diabetes and/or HbA1C > 6%. Additional exclusion criteria included ongoing pregnancy or lactation, >3 hospitalizations over the last 3 years and the use of any of the following within the last 3 months: systemic antibiotics, systemic, inhaled or intranasal corticosteroids or other immunosuppressants, cytokine therapy and/or large doses of pre/probiotic supplements. Use of tobacco products over the last 1 year (including electronic cigarettes) was also an exclusion criterion.

All study participants received a comprehensive oral examination, which included measurements of full mouth probing depth (PD), clinical attachment loss (CAL) and bleeding on probing (BOP) and panoramic radiography to determine periodontal status. All participants were screened for oral health, to exclude soft tissue lesions on the oral mucosa, active caries or acute periodontal/dental infections. Participants enrolled in the periodontitis group exhibited generalized inflammation (BOP > 10%), ≥4 interproximal sites with CAL ≥ 5 mm and radiographic bone loss. A total of 28 individuals met the inclusion criteria (Supplementary Table 1), consisting of 11 healthy individuals (all female; mean age 34.45 years) and 17 patients with periodontitis (7 female and 10 male; mean age 37.29 years).

#### Human gingiva biopsy collection

Oral biopsies of the gingiva were collected under local anesthesia from both healthy individuals and those with periodontitis. For the healthy group, biopsies were obtained from areas with no visible inflammation, no BOP and no radiographic evidence of bone loss. Periodontitis samples were collected from sites of active inflammation (BOP) and severe periodontal destruction (PD and CAL ≥ 5 mm). The exact orientation of each biopsy in relation to the tooth surface was recorded at collection and before processing. Biopsy materials that did not meet strict clinicopathologic criteria or had suboptimal orientation or tissue integrity were still included in the study but were excluded from direct quantitative comparisons between the health and periodontitis groups (Supplementary Table 1). Surgical tissue discards from periodontally involved sites obtained during periodontal surgeries were used for CITE-seq and spectral flow cytometry.

#### Human gingival sample preparation

Immediately following tissue acquisition, biopsy material was transferred to the laboratory, oriented and grossed into <2-mm pieces for processing according to the spatial transcriptomics (Xenium) and iterative immunostaining (IBEX) protocols. For Xenium, tissues were immersed in cryo-embedding compound (OCT) for 20 min in proper orientation at 25 °C followed by isopentane freezing. For IBEX, tissues were immersed in 1% paraformaldehyde for 72 h at 4 °C, followed by washing in 1× PBS and incubation in 30% sucrose at 4 °C overnight and were subsequently embedded in OCT.

Preparation of single-cell suspensions for CITE-seq and spectral flow cytometry was carried out as previously described^52^. In brief, tissue fragments were minced and digested using collagenase II (Worthington Biochemical Corporation) and DNase (Sigma) and dissociated with the gentleMACS Dissociator (Miltenyi). Cells were then passed through a 70-µm filter (Falcon, Corning), washed and counted (Cellometer Auto 2000, Nexcelom).

#### Spatial transcriptomics, Xenium (10x Genomics)

The 5-μm sections were placed on manufacturer-provided slides and stored at −80 °C for less than 4 weeks. Slides were then thawed for fixation, permeabilization and overnight probe hybridization followed by a post-hybridization wash, ligation, amplification, autofluorescence quenching and nuclei staining steps performed the next day. The Xenium analyzer workflow was initiated using a predesigned human multi-tissue panel (Human Multi-Tissue and Cancer) combined with a 76-plex custom probe panel (Supplementary Table 2). All steps were performed in accordance with manufacturer guidelines.

#### Human oral tissue iterative immunostaining, IBEX

The IBEX protocol was performed as previously described^53^ with minor modifications. In brief, 5-μm fixed-frozen tissue sections were placed directly onto a 25-mm round cover glass coated with 20 μl of chrome alum gelatin (Newcomer Supply). The cover glass was then adhered using low toxicity silicone elastomer material (World Precision Instruments) into a custom three-dimensional (3D) printed (Ultimaker3) chamber that allowed for consistent placement in the microscope stage insert. The chamber with the tissue sections was left to dry at room temperature inside a hood overnight. Sections were rehydrated with 1× PBS at room temperature for 5 min and blocked with 0.22 μm filtered 1× PBS containing 0.3% Triton-X-100 (VWR Life Sciences), 1% BSA (Sigma), 1% human Fc block (BD Biosciences) and 0.02% Hoechst (Biotium) at 37 °C for 1 h. Primary antibodies were incubated overnight at 4 °C, followed by secondary antibody incubation at 37 °C for 1 h, if applicable (Supplementary Table 3). The following antibodies were used: CK19 (unconjugated, mouse IgG2a, clone A53-B/A2; BioLegend 628502; 1:100 dilution), MCT (unconjugated, mouse IgG1, clone AA1; Dako M7052; 1:200 dilution), CK5 (unconjugated, guinea pig polyclonal; LS-Bio LS-C22715; 1:200 dilution), MPO (unconjugated, rabbit polyclonal; Abcam ab9535; 1:100 dilution), CD31-AF488 (mouse IgG1, clone WM59; BioLegend 303109; 1:50 dilution), Pan-CK-eF570 (mouse IgG1, clone AE1/AE3; Thermo Fisher 41-9003-80; 1:50 dilution), CD4-AF647 (mouse IgG1, clone RPA-T4; BioLegend 300523; 1:100 dilution), CD68-AF488 (mouse IgG2b, clone Y1/82A; BioLegend 333811; 1:100 dilution, not used in downstream analysis due to inconsistent quality), Ki67–eF570 (mouse IgG1, clone SolA15; Thermo Fisher 41-5698-80; 1:100 dilution), CD8α-F488 (mouse IgG1, clone RPA-T8; BioLegend 301024; 1:100 dilution), CD3-F594 (mouse IgG1, clone UCHT1; Caprico 1053134; 1:100 dilution), Thy1-AF647 (mouse IgG1, clone 5E10; BioLegend 328115; 1:100 dilution), S100A8/A9-AF488 (mouse IgG2a, clone 900028; R&D IC9337G; 1:100 dilution), α-SMA-eF570 (mouse IgG2a, clone 1A4; Thermo Fisher 41-9760-80; 1:100 dilution), CD45–iF594 (mouse IgG1, clone F10-89-4; Caprico 1016134; 1:100 dilution), CD138-AF647 (mouse IgG1, clone Syndecan-1; BioLegend 356523; 1:200 dilution), CD20-AF488 (mouse IgG2a, clone L26; Thermo Fisher 53-0202-82; 1:50 dilution), Vimentin–AF594 (mouse IgG2a, clone O91D3; BioLegend 677804; 1:600 dilution), HLA-DR-AF647 (mouse IgG2a, clone L243; BioLegend 307621; 4 °C O/N, 1:100 dilution), goat anti-mouse IgG2a AF488 (Thermo Fisher A21131; 1:500 dilution), goat anti-mouse IgG1AF555 (Thermo Fisher A21127; 1:500 dilution), goat anti-guinea pig AF647 (Thermo Fisher A21450; 1:500 dilution), goat anti-mouse IgG2b AF488 (Thermo Fisher A21141; 1:500 dilution) and donkey anti-rabbit AF647 (Jackson ImmunoResearch 711-606-152; 1:500 dilution).

Sections were equilibrated and imaged in water-soluble medium (Fluoromount G, Southern Biotech). Following image acquisition, the mounting medium was thoroughly washed off, fluorophore bleaching was performed using 1 mg ml^−1^ LiBH₄ (STREM Chemicals) diluted in dH_2_O for 30 min, and additional cycles of blocking and staining were performed. In subsequent cycles using the same isotypes as a previous indirectly labeled antibody, an additional blocking step was performed with highly concentrated (1:20 dilution) isotype controls for 1 h at 37 °C to block nonspecific antibody binding.

For visualization of HEVs, single cycle three-color immunofluorescence staining was carried out with 5-μm fixed-frozen sections placed on standard histology slides (Daigger Scientific) labeled with AF647-PNAd (clone MECA-79), iFluor594-CD3 (clone UCHT1) and AF488-CD31 (clone WM59).

#### IBEX image acquisition

IBEX images were acquired with a white-light laser confocal microscopy (Leica SP8) with a spectral output range between 470 nm and 670 nm equipped with an additional 405-nm laser line as well as three PMT and two HyD detectors. Images were acquired with a ×20 objective (0.75 NA). Pixel dimensions were 0.444 × 0.444 μm with a 16-bit depth. Sequential imaging between lines and bidirectional imaging were enabled. A *z*-stack containing the brightest slice (*z*-step range, 0.6–1.3 μm) was obtained. Raw outputs were then launched in LAS X software and the *z* slice that had the highest signal to noise ratio for all channels was selected and cropped to generate a two-dimensional image for all cycles of each sample. Huygens v.23.04 (Scientific Volume Imaging, http://svi.nl) was used for stitching of multi-tile images. Using Imaris File Converter, multichannel files from each channel were then converted to Imaris format, renamed with XTConfigureChannelSettings and registered based on Hoechst with XTRegisterSameChannel^54^.

#### IBEX image pre-processing and object-based segmentation

Registered multichannel images, were processed automatically in Fiji^55^. First, tissue areas that were partially unregistered or detached in any cycle were removed from all channels and all cycles via automated selection of nonoverlapping Hoechst masks in addition to manual exclusion of gross unregistered areas. Then, each individual channel was selected and processed with median filter, background subtraction, thresholding and small object elimination. For small object elimination, a mask that included positive pixels (above threshold) but also eliminated small objects for each channel (smaller than the minimum truly positive cell) was generated using the ‘Analyze Particles’ function. The mask region of interest (ROI) was superimposed to the original image and every signal outside of this ROI was removed from the channel of interest. All parameters were selected individually for each channel and kept constant across all samples and experimental batches.

For object-based segmentation, we used a combination of Fiji, Ilastik^56^ and Cellprofiler^57^. In brief, two-channel images were generated in Fiji, with the nuclei channel derived from the final cycle Hoechst channel and the cell border channel created from a composite of all membrane and cytoplasmic markers using unprocessed images (SimpleITK registration output). The two-channel stacks were then imported in Ilastik where a pixel classification project was created. Training was performed based on all samples until the probability maps for nuclei, cell borders and background were histologically accurate, and the uncertain regions were minimal. Ilastik outputs were then imported to CellProfiler to generate the segmentation masks which were used for the subsequent analysis.

#### IBEX cell phenotyping

Per-channel protein intensities were quantified at the single-cell level using MCQuant^58^ and clustering was performed using scimap^59^, with intensity values scaled via the ‘rescale’ function and automatic gating applied using a Gaussian mixture model. Initial clustering was carried out using the ‘phenotype_cells’ function with user-defined marker combinations, followed by subclustering within each phenotype using the *k*-means algorithm. When appropriate, clustering was restricted to a subset of relevant markers. For more accurate clustering of epithelial subregions, pathologist annotations were incorporated using the ‘addROI_image’ tool.

#### Xenium data processing and analysis

All default Xenium segmentation outputs were re-segmented using the command ‘resegment’ with an expansion distance set to 0 within the Xenium Ranger software. Xenium regions containing more than one tissue section were cropped using a custom function, such that each resulting region contained a single tissue section. In brief, a region of interest was drawn by hand in Xenium Explorer, and the region coordinates were exported as a comma-separated value file. These coordinates were used to modify the relevant raw Xenium data (cell_boundaries.csv, cells.csv, transcripts.csv and barcodes.csv) to include only coordinates within the region of interest.

Individual sections were merged into a single Seurat^60^ object, preserving section-specific metadata. This object was then split into individual layers representing each region. Individual regions underwent normalization followed by standard dimensionality reduction (Seurat functions: NormalizeData, FindVariableFeatures, ScaleData and RunPCA). Integration of all sections was performed using ‘IntegrateLayers’ using the RPCAIntegration method. Clustering and cluster marker genes were performed using Seurat functions (FindNeighbors, FindClusters and FindAllMarkers). Cells lacking distinct gene expression, or gene expression inconsistent with known cell types, were labeled ‘unknown’ and removed from analysis. Seurat objects were converted to AnnData format for further analysis.

#### Neighborhood analysis for IBEX and Xenium datasets

Spatial niche analysis was performed using the ‘spatial_expression’ tool from scimap to generate weighted neighborhood matrices for both the IBEX and Xenium datasets. Neighborhoods were defined using a *k*-nearest neighbors (*k-*NN) approach. The resulting neighborhood expression matrices were then analyzed with the ‘spatial_cluster’ tool, using *k*-means clustering as the dimensionality reduction method. A gradient of neighborhood sizes (number of nearest neighbors) and a range of cluster numbers (*k*) were applied iteratively to optimize niche resolution. For final clustering, 30 nearest neighbors were used for IBEX and 50 for Xenium, reflecting differences in tissue architecture and spatial resolution. When necessary, niche clusters were manually merged based on shared cell-type composition and histologic context.

#### Spatial proximity analysis of immune niches to TAE

To assess the spatial proximity of immune cell niches to the TAE niche, a custom Python script was used, utilizing scikit-learn for pairwise Euclidean distance calculations between cells based on their *X* and *Y* centroids. The analysis was restricted to sections with ≥200 TAE cells to ensure robust results. Pairwise distances were calculated between TAE and immune cells. A maximum distance threshold of 3,000-pixel units was applied to account for variation in section size and to exclude distant areas that appeared only in larger sections. For each image and niche, the analysis quantified (1) the number of immune cells within the 3,000-unit threshold and (2) the average distance between TAE and immune niche cells.

#### CITE-seq

Single-cell preparations of between 5 × 10^4^ and 5 × 10^5^ single cells from periodontitis samples were stained individually with TotalSeq-A Human Universal Cocktail, v.1.0 (BioLegend) as per the manufacturer’s protocol (Supplementary Table 4). The 1.7 × 10^4^ stained cells were then loaded with the reverse transcription mix on the Chromium chip G and partitioned into single cells in gel beads-in-emulsion (GEMs) using the 10x Genomics Chromium Next GEM Single Cell 3’ kit v3.1, as per the manufacturer’s instructions (10x Genomics). Complementary DNA amplification and ADT libraries were prepared as per instructions from BioLegend, and gene expression libraries were made as per the 10x Genomics protocol. All amplification steps were performed in an Applied Biosystems Instruments Veriti 96-well thermal cycler. Quality and quantity of the libraries were assessed using TapeStation (Agilent) and a Qubit fluorometer (Thermo Fisher). Libraries were pooled at a concentration of 2 nM and sequenced on an Illumina platform (NextSeq 2000, Illumina) using the read lengths as read 1, 28 bp; index 1, 10 bp; index 2, 10 bp; and read 2, 90 bp.

Processing of CITE-seq data was performed in R using DecontX to correct for ambient RNA, DSB to normalize and denoise ADT counts and DoubletFinder to remove putative doublets. Seurat was used for data normalization, determination of variable features, data scaling and principal component analysis (PCA). RPCA integration was performed on RNA and ADT assays and a WNN graph was created using the Seurat FindMultiModalNeighbors. General cell types were identified using FindClusters and FindAllMarkers functions before subsetting the integrated data by cell type. Variable feature identification, scaling, PCA, RPCA integration and WNN graph creation were then performed within each cell type to identify cell type subsets.

#### Spectral flow cytometry

Freshly isolated oral tissue samples yielding between 7 × 10^5^ and 2 × 10^6^ single cells were processed for flow cytometric analysis using a 36-color panel (Supplementary Table 5). Samples were first stained with Live/Dead reagent for 15 min at 25 °C, washed and incubated with Fc block for 10 min at 25 °C. Cells were next incubated with a pool of six panel antibodies for 10 min at 25 °C: CCR5, CCR6, CCR7, CXCR3, CXCR5 and TCRgd. A pool of all remaining panel antibodies was then added and samples incubated for additional 30 min at 25 °C. Following this, cells were washed and fixed with 1% paraformaldehyde for 10 min at 25 °C. Acquisition was conducted using a Cytek Aurora with spectral unmixing and autofluorescence extraction using SpectroFlo software. For unmixing, single color reference controls were used of each antibody staining beads (UltraComp eBeads Plus, Thermo Fisher) and viability dye staining in a mixture of live and heat-killed peripheral blood mononuclear cells. For autofluorescence extraction, controls of unstained matching oral tissue sample were used. Data analysis was performed in FlowJo.

#### CITE-seq and Xenium integration for spatial imputation of cluster identities

Computational integration between CITE-seq and Xenium data was performed to spatially impute fine-grained cluster identities from CITE-seq into Xenium samples. In brief, the CITE-seq Seurat objects were converted to AnnData format. Xenium and CITE-seq AnnData objects were subset into broad cellular compartments (epithelial, stromal and immune) and each compartment was processed independently. Each dataset was concatenated, scaled and underwent PCA, followed by batch correction using the ‘scanpy.external.pp.harmony_integrate’ function. Cell-type labels from CITE-seq were then transferred to Xenium using *k*-NN classification based on the harmonized PCA embeddings. Finally, the integrated epithelial, stromal and immune datasets were merged into a single AnnData object for downstream analysis.

#### Barrier epithelial cell cross-organ integration and reanalysis

Gingival epithelial cells from the human oral single-cell atlas^13^ were integrated with epithelial cells from the Xenium dataset for label transfer from the Xenium to the single-cell dataset. Label-transferred epithelial cells from healthy gingiva were then concatenated with epithelial cells from the Tabula Sapiens reference atlas^17^ for cross-organ comparison. The merged dataset was then normalized, log-transformed and reduced to the top 2,000 highly variable genes. Data rescaling and PCA were performed, followed by batch correction using ‘scanpy.external.pp.harmony_integrate’. For cross-organ comparison, the integrated dataset was further filtered to include only epithelial cells from mucosal barrier tissues (oral mucosa, gastrointestinal tract and lung), while excluding nonbarrier epithelial subsets, such as secretory or neuroendocrine cells. The top 30 defining genes for each organ were submitted to Enrichr^51^ to identify the most enriched Gene Ontologies specific to each mucosal barrier tissue.

#### Bacterial FISH and immunofluorescence labeling on human gingival tissues

FISH was performed using eubacterial FISH probe (GCTGCCTCCCGTAGGAGT) conjugated to rhodamine-red X fluorophore (Integrated DNA Technologies) on 5-μm fixed-frozen sections. Hybridization was carried out at 46 °C O/N in hybridization buffer (0.09 M NaCl, 0.02 M Tris, pH 7.5, 0.01% SDS and 20% formamide) and 2 µM EUB probe. Slides were washed in wash buffer 1 (0.09 M NaCl, 0.02 M Tris, pH 7.5, 0.01% SDS and 20% formamide) for 15 min at 48 °C followed by three washes in wash buffer 2 (0.09 M NaCl, 0.02 M Tris, pH 7.5 and 0.01% SDS) for 15 min at 48 °C. Slides were dehydrated in an ethanol series before proceeding to immunofluorescence labeling. For CK19 immunofluorescence labeling, the sections were permeabilized for 10 min with PBS containing 0.1 % Tween 20. Followed by three PBS washes. To block nonspecific binding of antibody, sections were incubated with 1% BSA, 22.52 mg ml^−1^ glycine in PBST for 30 min. Sections were then incubated in the diluted (1:100) primary antibody (clone A53-B/A2) for 1 h at 25 °C. The slides were washed three times for 5 min each with PBS. The sections were further incubated with the anti-mouse IgG2a conjugated to AF488 (1:100) for 1 h in the dark. The slides were washed three times for 5 min each with PBS. The sections were then counter stained with 3.0 μg ml^−1^ 4,6-diamidino-2-phenylindole (DAPI) for 5 min and rinsed with PBS. The slides were mounted in Prolong Gold antifade medium (Thermo Fisher) and allowed to cure for at least 24 h before imaging.

Images were acquired using the LSM 980 confocal microscope (Carl Zeiss). The spectral images were acquired with the Plan-Apochromat ×20/0.8 NA for a full tile image of the entire tissue field of view and Plan-Apochromat ×63/1.4 NA for hi-res of regions of interest. The 24-channel spectral images were acquired by simultaneous excitation with 488 nm and 561 nm lasers and signals collected from 490–695 nm. The 405 nm laser was used independently to image DAPI in a separate image. The 3D z-stack images were acquired with 7–9 z-planes up to a thickness of 7 μm. Spectral unmixing was performed in Zenv v.3.6 and image brightness and contrast were adjusted for each unmixed channel separately in Fiji ImageJ v.1.54F.

#### Statistics and reproducibility

No statistical methods were used to predetermine sample sizes, but our sample sizes are similar to or exceed those reported in previous publications^13,17,43^. Unless otherwise indicated, statistical comparisons were performed using nonparametric tests, as the datasets did not meet the assumptions of normality required for parametric testing. Normality and equal variance were not formally tested.

All selected photomicrographs are representative of spatial patterns reproducibly observed across all appropriately oriented samples and experimental batches (spatial proteomics, *n* = 8 (oral health) and *n* = 8 (periodontitis) participants across three independent experiments; spatial transcriptomics, *n* = 8 (oral health) and *n* = 11 (periodontitis) participants across two independent experiments).

Data collection and analysis were not performed blind to the conditions of the experiments, but analysis was performed using automated and unsupervised methods. No subjective input influenced the analysis outputs. No randomization occurred during sample collection or analysis.

### Reporting summary

Further information on research design is available in the Nature Portfolio Reporting Summary linked to this article.

## Online content

Any methods, additional references, Nature Portfolio reporting summaries, source data, extended data, supplementary information, acknowledgements, peer review information; details of author contributions and competing interests; and statements of data and code availability are available at 10.1038/s41590-025-02398-y.

## Supplementary information

Reporting Summary

Supplementary Table 1 Human participant metadata.

Supplementary Table 2 Xenium gene list.

Supplementary Table 3 IBEX cycles.

Supplementary Table 4 Total_Seq_Cocktail.

Supplementary Table 5 Spectral_flow_panel.

## Source data

Source Data Fig. 1 Numerical source data.

Source Data Fig. 2 Numerical source data.

Source Data Fig. 3 Numerical source data.

Source Data Fig. 4 Numerical source data.

Source Data Fig. 5 Numerical source data.

Source Data Fig. 6 Numerical source data.

Source Data Extended Fig. 1 Numerical source data.

Source Data Extended Fig. 2 Numerical source data.

Source Data Extended Fig. 3 Numerical source data.

Source Data Extended Fig. 4 Numerical source data.

Source Data Extended Fig. 5 Numerical source data.

Source Data Extended Fig. 6 Numerical source data.

Source Data Extended Fig. 7 Numerical source data.

## Data Availability

Raw and processed CITE-seq datasets have been deposited in the NCBI Gene Expression Omnibus database under accession code GSE296447. Xenium and IBEX raw datasets have been deposited in Harvard Dataverse and can be accessed at 10.7910/DVN/3PFU0D and 10.7910/DVN/F2ICVP. Source data are provided with this paper.

## Code availability

Code to reproduce findings in this paper is available on Github and may be accessed at https://github.com/dwilliamsLab/gingiva-2025.
