# Supplementary material for: Distinct spatial organization governs oral mucosal immunity
Source: Nat Immunol. 2026 Feb 9;27(3):624–35. doi: 10.1038/s41590-025-02398-y (PMC12956602; doi:10.1038/s41590-025-02398-y)
Supplement: Supplementary file 1 — Reporting Summary [file 41590_2025_2398_MOESM1_ESM.pdf]

nature portfolio

Corresponding author(s): Drake Williams, Niki Moutsopoulos

Last updated by author(s): Nov 25, 2025

Reporting Summary

Nature Portfolio wishes to improve the reproducibility of the work that we publish. This form provides structure for consistency and transparency in reporting. For further information on Nature Portfolio policies, see our Editorial Policies and the Editorial Policy Checklist.

Statistics

For all statistical analyses, confirm that the following items are present in the figure legend, table legend, main text, or Methods section.

n/a	Confirmed	
☐	☒	The exact sample size (*n*) for each experimental group/condition, given as a discrete number and unit of measurement
☐	☒	A statement on whether measurements were taken from distinct samples or whether the same sample was measured repeatedly
☐	☒	The statistical test(s) used AND whether they are one- or two-sided<br>*Only common tests should be described solely by name; describe more complex techniques in the Methods section.*
☒	☐	A description of all covariates tested
☐	☒	A description of any assumptions or corrections, such as tests of normality and adjustment for multiple comparisons
☐	☒	A full description of the statistical parameters including central tendency (e.g. means) or other basic estimates (e.g. regression coefficient) AND variation (e.g. standard deviation) or associated estimates of uncertainty (e.g. confidence intervals)
☐	☒	For null hypothesis testing, the test statistic (e.g. *F*, *t*, *r*) with confidence intervals, effect sizes, degrees of freedom and *P* value noted<br>*Give P values as exact values whenever suitable.*
☒	☐	For Bayesian analysis, information on the choice of priors and Markov chain Monte Carlo settings
☒	☐	For hierarchical and complex designs, identification of the appropriate level for tests and full reporting of outcomes
☒	☐	Estimates of effect sizes (e.g. Cohen's *d*, Pearson's *r*), indicating how they were calculated

Our web collection on statistics for biologists contains articles on many of the points above.

Software and code

Policy information about availability of computer code

Data collection	For spatial proteomic data acquisition, Leica SP8 confocal microscope with a spectral output range between 470nm and 670nm equipped with an additional 405nm laser line as well as 3 PMT and 2 HyD detectors. Images were collected with LAS X software.<br>For the spatial transcriptomics, data were acquired in Xenium Analyzer equipped with the Xenium Explorer 2.0 software.<br>Spectral flow cytometry data were collected in Cytek Aurora using SpectroFlo software.<br>Citeseq libraries were generated using the 10X Genomics Chromium Next GEM Single Cell 3’ Kit v3.1 Sequencing was performed on an Illumina NextSeq 2000 system.
Data analysis	For spatial proteomic data processing the following tools were used: LAS X v3.7.4(brightest section selection), Huygens v23.04 (image stitching), Imaris File Converter v9.5 (file conversion to imaris format), SimpleITK toolkits XTConfigureChannelSettings (channel renaming) and XTRegisterSameChannel (image registration), ImageJ v1.54p (removal of detached areas, automated channel processing and area calculation), Ilastik 1.4.0 (probability map generation) and Cellprofiler v4.2.1 (cell segmentation).<br>Spatial transcriptomic data analysis was conducted in R v 4.3.1 with Seurat 5.0.1, as well as in Python v3.8.17 with scimap v1.3.1, scanpy v1.9.1, scikit-learn v1.3.0, scipy v1.9.1, anndata v0.9.2, pandas v1.5.3, seaborn 0.11.2, numpy v1.22.4.<br>Spatial proteomic data analysis was performed in Python v3.8.17 using scimap v1.3.1, scanpy v1.9.1, scikit-learn v1.3.0, scipy v1.9.1, anndata v0.9.2, pandas v1.5.3, seaborn 0.11.2, numpy v1.22.4.<br>CITE-seq analysis was performed using the R platform (4.4.3) with R Studio (2023.12.1+402), Seurat (5.2), and tidyverse (2.0.0)<br>Integration between different datasets was conducted in Python v3.10.16 using scanpy v1.10.4.<br>Images were generated using ImageJ v1.54p, matplotlib v3.10.0, ggplot2 v3.5.0, and Adobe Illustrator v29.5.1.

For manuscripts utilizing custom algorithms or software that are central to the research but not yet described in published literature, software must be made available to editors and reviewers. We strongly encourage code deposition in a community repository (e.g. GitHub). See the Nature Portfolio guidelines for submitting code & software for further information.

Data

Policy information about availability of data

All manuscripts must include a data availability statement. This statement should provide the following information, where applicable:

- Accession codes, unique identifiers, or web links for publicly available datasets
- A description of any restrictions on data availability
- For clinical datasets or third party data, please ensure that the statement adheres to our policy

Raw and processed CITE-seq datasets have been deposited in the NCBI GEO database under accession GSE296447 and can been accessed via the following link: https://www.ncbi.nlm.nih.gov/geo/query/acc.cgi?acc=GSE296447. Xenium and IBEX raw datasets have been deposited in Harvard Dataverse and can be accessed via the following links: https://doi.org/10.7910/DVN/3PFU0D and https://doi.org/10.7910/DVN/F2ICVP

Code to reproduce findings in this manuscript is available on Github and may be accessed here: https://github.com/dwilliamsLab/gingiva-2025.

Research involving human participants, their data, or biological material

Policy information about studies with human participants or human data. See also policy information about sex, gender (identity/presentation), and sexual orientation and race, ethnicity and racism.

Reporting on sex and gender	Information on sex (self reporting) for all participants is located in Supplemental Table 1. Information on gender not gathered/reported
Reporting on race, ethnicity, or other socially relevant groupings	Reporting on race for all participants (based on self reporting) can be found in Supplemental Table 1.
Population characteristics	While demographic information is captured (age, sex, race) study sample numbers do not allow for sub classification of the population based on these variables. Main variable by which study cohort was categorized is oral health versus severe periodontal disease (based on comprehensive clinical evaluation).
Recruitment	Participant recruitment included self referral (for healthy subjects) and self referral as well as practitioner referral for cases of severe untreated periodontal disease. Broad dissemination of recruitment flyers (through social media outlets and NIH patient services)aimed to reduce bias in selection - particularly of healthy volunteers. Patient volunteer recruitment relied more heavily on dentist/practitioner referrals.
Ethics oversight	Office of Human Subjects Research Protection (OHSRP), Intramural program of the NIH

Note that full information on the approval of the study protocol must also be provided in the manuscript.

Field-specific reporting

Please select the one below that is the best fit for your research. If you are not sure, read the appropriate sections before making your selection.

☒ Life sciences ☐ Behavioural & social sciences ☐ Ecological, evolutionary & environmental sciences

For a reference copy of the document with all sections, see nature.com/documents/nr-reporting-summary-flat.pdf

Life sciences study design

All studies must disclose on these points even when the disclosure is negative.

Sample size	Sample size was determined based on feasibility, data quality, and the technical limitations of high-dimensional spatial omics approaches. A total of 28 human subjects (17 periodontitis and 11 healthy) were included. The chosen sample size exceeds those used in comparable published studies and can be sufficient for generating meaningful insights in exploratory multi-omic tissue studies.
Data exclusions	Exclusion criteria of individual samples for the spatial transcriptomic and proteomic analysis are detailed in supplementary table 1.
Replication	Spatial proteomics experiments were conducted in three independent experimental batches, each containing a balanced and randomly assigned representation of healthy and periodontitis samples to minimize batch-related bias. Spatial transcriptomics was performed in two batches using an automated workflow following the manufacturer's (10x Genomics) recommended protocol. Results were consistent across batches for both spatial proteomics and spatial transcriptomics experiments.
Randomization	Samples were randomly assigned to experimental batches to ensure equal representation of healthy and periodontitis groups across batches. No other randomization was applicable.
Blinding	Data analysis was fully automated and unsupervised; all computational tools operated without access to the human subject condition. No subjective input influenced the analysis outputs.

Reporting for specific materials, systems and methods

We require information from authors about some types of materials, experimental systems and methods used in many studies. Here, indicate whether each material, system or method listed is relevant to your study. If you are not sure if a list item applies to your research, read the appropriate section before selecting a response.

Materials & experimental systems

n/a	Involved in the study
☐	☒ Antibodies
☒	☐ Eukaryotic cell lines
☒	☐ Palaeontology and archaeology
☒	☐ Animals and other organisms
☐	☒ Clinical data
☒	☐ Dual use research of concern
☒	☐ Plants

Methods

n/a	Involved in the study
☒	☐ ChIP-seq
☐	☒ Flow cytometry
☒	☐ MRI-based neuroimaging

Antibodies

Antibodies used

Spatial proteomics antibodies (Supplementary Table 3)

CK19 [unconjugated - mouse IgG2a, clone A53-B/A2; BioLegend 628502; 4°C O/N, 1:100], MCT [unconjugated - mouse IgG1, clone AA1; Dako M7052; 4°C O/N, 1:200], CK5 [unconjugated - guinea pig polyclonal; LS-Bio LS-C22715; 4°C O/N, 1:200], MPO [unconjugated - rabbit polyclonal; Abcam ab9535; 4°C O/N, 1:100], CD31-AF488 [mouse IgG1, clone WM59; BioLegend 303109; 4°C O/N, 1:50], Pan-CK-eF570 [mouse IgG1, clone AE1/AE3; ThermoFisher 41-9003-80; 4°C O/N, 1:50], CD4-AF647 [mouse IgG1, clone RPA-T4; BioLegend 300523; 4°C O/N, 1:100], CD68-AF488 [mouse IgG2b, clone Y1/82A; BioLegend 333811; 4°C O/N, 1:100], Ki-67–eF570 [mouse IgG1, clone SolA15; ThermoFisher 41-5698-80; 4°C O/N, 1:100], CD8α-F488 [mouse IgG1, clone RPA-T8; BioLegend 301024; 4°C O/N, 1:100], CD3-F594 [mouse IgG1, clone UCHT1; Caprico 1053134; 4°C O/N, 1:100], Thy1-AF647 [mouse IgG1, clone 5E10; BioLegend 328115; 4°C O/N, 1:100], S100A8/A9-AF488 [mouse IgG2a, clone 900028; R&D IC9337G; 4°C O/N, 1:100], α-SMA-eF570 [mouse IgG2a, clone 1A4; ThermoFisher 41-9760-80; 4°C O/N, 1:100], CD45–iF594 [mouse IgG1, clone F10-89-4; Caprico 1016134; 4°C O/N, 1:100], CD138-AF647 [mouse IgG1, clone Syndecan-1; BioLegend 356523; 4°C O/N, 1:200], CD20-AF488 [mouse IgG2a, clone L26; ThermoFisher 53-0202-82; 4°C O/N, 1:50], Vimentin–AF594 [mouse IgG2a, clone O91D3; BioLegend 677804; 4°C O/N, 1:600], HLA-DR-AF647 [mouse IgG2a, clone L243; BioLegend 307621; 4°C O/N, 1:100], Goat anti-mouse IgG2a AF488 [(ThermoFisher A21131); 1 h, 37°C, 1:500], Goat anti-mouse IgG1AF555 [(ThermoFisher A21127); 1 h, 37°C, 1:500], Goat anti-guinea pig AF647 [(ThermoFisher A21450); 1 h, 37°C, 1:500], Goat anti-mouse IgG2b AF488 [(ThermoFisher A21141); 1 h, 37°C, 1:500], Donkey anti-rabbit AF647 [(Jackson ImmunoResearch 711-606-152); 1 h, 37°C, 1:500]

Spectral flow cytometry antibodies (Supplementary Table 5)

CD45RA–BUV395 [clone 5H9; BD 740315],CD16–BUV496 [clone 3G8; BD 612944], CCR5–BUV563 [clone 2D7/CCR5; BD 741401], CD11c–BUV661 [clone B-Ly6; BD 612967], C D56–BUV737 [clone NCAM16.2; BD 612766], CD8–BUV805 [clone SK1; BD 612889], Viability–LIVE/DEAD Blue [Thermo L34962], CCR7 (CD197)–BV421 [clone G043H7; BioLegend 353208], CD123–Super Bright 436 [clone 6H6; Thermo 62-1239-42], CD161–eFluor450 [clone HP-3G10; Thermo 48-1619-42], IgD–BV480 [clone IA6-2; BD 566138], CD3–BV510 [clone SK7; BioLegend 344828], CD20–Pacific Orange [clone HI47; Thermo MHCD2030], IgM–BV570 [clone MHM-88; BioLegend 314517], IgG–BV605 [clone G18-145; BD 563246], CD28–BV650 [clone CD28.2; BioLegend 302946], CCR6 (CD196)–BV711 [clone G034E3; BioLegend 353436], CXCR5 (CD185)–BV750 [clone RF8B2; BD 747111], PD-1 (CD279)–BV785 [clone EH12.2H7; BioLegend 329929], CD15–BB515 [clone HI98; BD 565236], CD57–FITC [clone HNK-1; BioLegend 359604], CD14–Spark Blue 550 [clone 63D3; BioLegend 367148], CD45–PerCP [clone H130; Thermo MHCD4531], CD11b–PerCP-Cy5.5 [clone ICRF44; BioLegend 301328], TCRγδ–PerCP-eFluor710 [clone B1.1; Thermo 46-9959-42], CD25–PE [clone BC96; Thermo 12-0259-42], CD4–CF568 (cFluor YG584) [clone SK3; Cytek R7-20041], CD66b–PE/Dazzle594 [clone G10F5; BioLegend 305122], CD95 (Fas)–PE-Cy5 [clone DX2; Thermo 15-0959-42], CXCR3 (CD183)–PE-Cy7 [clone CEW33D; Thermo 25-1839-42], CD27–APC [clone O323; Thermo 17-0279-42], CD1c–Alexa Fluor 647 [clone L161; BioLegend 331510], CD19–Spark NIR 685 [clone HIB19; BioLegend 302270], CD127–APC-R700 [clone HIL-7R-M21; BD 565185], HLA-DR–APC/eFluor780 [clone L243; Thermo 47-9952-42], CD38–APC/Fire810 [clone HIT2; BioLegend 303550].

Validation

Each antibody used in the spatial proteomics workflow was independently validated through titration experiments (ranging from 1:50 to 1:1000), and the dilution that yielded the optimal signal-to-noise ratio was selected. Antibodies were subsequently tested in multiplexed panels within each IBEX imaging cycle to confirm compatibility and to ensure there was no signal crosstalk or other adverse effects. Due to the unique technical characteristics of oral tissues, internal validation was necessary for all antibodies to ensure robust performance in our specific tissue context.

Clinical data

Policy information about clinical studies

All manuscripts should comply with the ICMJE guidelines for publication of clinical research and a completed CONSORT checklist must be included with all submissions.

Clinical trial registration

ClinicalTrials.gov ID NCT01568697

Study protocol	ClinicalTrials.gov
Data collection	This is a cross sectional/natural history protocol with at minimum a screening visit and a study visit. Clinical Data are collected during the screening and study visits
Outcomes	Primary Objectives<br><br>Investigate the degree, scope, etiology and natural history of oral manifestations in patients with genetic immune defects.<br>Characterize the immune response in the oral cavity of patients with genetic immune defects.<br>Characterize the microbiome in the oral cavity of patients with genetic immune defects.<br><br>Secondary Objectives<br><br>Assay development/validation for the study of tissue immunity and microbiome characterization<br>Establishment of normative values for immune mediators and microbial elements at the oral cavity<br><br>Primary Outcome Measures<br><br>Clinical intraoral characterization (i.e., presence and severity of periodontitis).<br>Characterize the immune response in the oral cavity of patients with genetic immune defects<br>Characterize the microbiome in the oral cavity of patients with genetic immune defects<br>Secondary Outcome Measures<br><br>Assay development/validation for the study of tissue immunity and microbiome characterization<br>Establishment of normative values for immune mediators and microbial elements at the oral cavity

Plants

Seed stocks	*Report on the source of all seed stocks or other plant material used. If applicable, state the seed stock centre and catalogue number. If plant specimens were collected from the field, describe the collection location, date and sampling procedures.*
Novel plant genotypes	*Describe the methods by which all novel plant genotypes were produced. This includes those generated by transgenic approaches, gene editing, chemical/radiation-based mutagenesis and hybridization. For transgenic lines, describe the transformation method, the number of independent lines analyzed and the generation upon which experiments were performed. For gene-edited lines, describe the editor used, the endogenous sequence targeted for editing, the targeting guide RNA sequence (if applicable) and how the editor was applied.*
Authentication	*Describe any authentication procedures for each seed stock used or novel genotype generated. Describe any experiments used to assess the effect of a mutation and, where applicable, how potential secondary effects (e.g. second site T-DNA insertions, mosiacism, off-target gene editing) were examined.*

Flow Cytometry

Plots

Confirm that:

☒ The axis labels state the marker and fluorochrome used (e.g. CD4-FITC).

☒ The axis scales are clearly visible. Include numbers along axes only for bottom left plot of group (a 'group' is an analysis of identical markers).

☒ All plots are contour plots with outliers or pseudocolor plots.

☒ A numerical value for number of cells or percentage (with statistics) is provided.

Methodology

Sample preparation	For spectral flow cytometry periodontitis tissue fragments were minced and digested using collagenase II (Worthington Biochemical Corporation) and DNase (Sigma) and dissociated with the gentleMACS Dissociator (Miltenyi). Cells were then passed through a 70mm filter (Falcon, Corning), washed, and counted (Cellometer Auto 2000 - Nexcelom).
Instrument	Acquisition was conducted using a Cytek Aurora with spectral unmixing and autofluorescence extraction using SpectroFlo software
Software	Spectral flow cytometry analysis was carried out in FlowJo software.
Cell population abundance	Populations displayed were fairly abundant (no rare populations reported on) and were largely confirmatory for the CITEseq data. Immune cell subpopulations displayed were gated within the B/Plasma cell fraction and within the antigen presenting cell compartments

Gating strategy

The exact gating strategy for the spectral flow cytometry is shown in Extended Figure 5.

☒ Tick this box to confirm that a figure exemplifying the gating strategy is provided in the Supplementary Information.
